# Evolutionary action and structural basis of the allosteric switch controlling β_2_AR functional selectivity

**DOI:** 10.1038/s41467-017-02257-x

**Published:** 2017-12-18

**Authors:** Anne-Marie Schönegge, Jonathan Gallion, Louis-Philippe Picard, Angela D. Wilkins, Christian Le Gouill, Martin Audet, Wayne Stallaert, Martin J. Lohse, Marek Kimmel, Olivier Lichtarge, Michel Bouvier

**Affiliations:** 1 Department of Biochemistry, Institute for Research in Immunology and Cancer, Université de Montreal, Montreal, QC Canada; 2 Department of Structural and Computational Biology and Molecular Biophysics, Baylor College of Medicine, Houston, TX USA; 3 Department of Molecular and Human Genetics, Baylor College of Medicine, Houston, TX USA; 4 Institute of Pharmacology and Toxicology, Würzburg, Germany; 5 Max Delbrück Center for Molecular Medicine, Berlin, Germany; 6 Departments of Statistics and Bioengineering, Rice University, Houston, TX USA

## Abstract

Functional selectivity of G-protein-coupled receptors is believed to originate from ligand-specific conformations that activate only subsets of signaling effectors. In this study, to identify molecular motifs playing important roles in transducing ligand binding into distinct signaling responses, we combined in silico evolutionary lineage analysis and structure-guided site-directed mutagenesis with large-scale functional signaling characterization and non-negative matrix factorization clustering of signaling profiles. Clustering based on the signaling profiles of 28 variants of the β_2_-adrenergic receptor reveals three clearly distinct phenotypical clusters, showing selective impairments of either the Gi or βarrestin/endocytosis pathways with no effect on Gs activation. Robustness of the results is confirmed using simulation-based error propagation. The structural changes resulting from functionally biasing mutations centered around the DRY, NPxxY, and PIF motifs, selectively linking these micro-switches to unique signaling profiles. Our data identify different receptor regions that are important for the stabilization of distinct conformations underlying functional selectivity.

## Abstract (web summary)

Ligand-induced biased signaling is thought to result in part from ligand-specific receptor conformations that cause the engagement of distinct effectors. Here the authors trace and evaluate the impact of mutations of the β2–adrenergic receptor on multiple signaling outputs to provide structural-level insight into the determinants of GPCR functional selectivity.

## Introduction

G-protein-coupled receptors (GPCR) form the largest family of receptors involved in cellular signaling. Diverse external stimuli such as hormones, neurotransmitters, metabolites, ions, and fatty acids are translated into a cellular response via GPCR activation. The wide range of GPCR-regulated cellular responses and disease associations with GPCR dysregulation make this system a prime target for drug discovery and development. In recent years, it has become evident that a single GPCR can couple not only to one but to several different G-protein subtypes leading to complex signaling profiles^1,2^. In addition, G-protein-independent signaling of GPCRs has been reported; primarily through βarrestins, which are also part of the desensitization/endocytosis machinery^3,4^. It follows that individual GPCRs have greater repertoires of cognate downstream signaling partners than originally anticipated, making their signaling more akin to a network than to a linear cascade. Furthermore, ligands have been identified that bind to the same GPCR but activate distinct and specific subsets of potential signaling pathways^5–9^. This phenomenon, called ligand-biased signaling or functional selectivity, has important implication for drug discovery as it paves the way for the identification of ligands that selectively target signaling pathways with therapeutic relevance while sparing pathways that could underlie undesirable effects. Currently, however, the possibility to rationally design compounds with intended signaling profiles is limited by the poorly understood molecular and structural determinants of functional selectivity.

Translating extracellular ligand binding to intracellular signaling relies on numerous small structural rearrangements of receptors, and functional selectivity is believed to result from differences in these rearrangements. Structural changes accompanying GPCR activation include an elongation and rotation of TM5, an outward movement of TM6 and an inward movement of TM7^10–13^. It is thought that these conformational changes are mediated by a conserved network of non-covalent contacts and that these allosteric rearrangements define activation pathways^14–17^. Such activation pathways include small groups of structurally neighboring amino acids within the seven-helical transmembrane domain common to GPCR that are called microswitches. These include the DRY motif (D130^3.49^, R131^3.50^, Y132^3.51^), the toggle switch (W286^6.48^), the NPxxY motif (N322^7.49^, P323^7.50^, Y326^7.53^)^18–20^, and the PIF/connector motif (P211^5.50^, I121^3.40^, F282^6.44^)^13,21^. However, in recent years, molecular dynamic simulations and structural studies of GPCRs in solution have shown that GPCR conformations are very dynamic and ligand dependent^22–26^. These observations suggest that different ensembles of receptor conformations might engage different effectors and thereby induce functional selectivity.

Mutations in GPCRs, including both synthetic and naturally occurring ones associated to diseases, have been shown to selectively alter subsets of the signaling repertoire^27–29^. Such mutagenesis-directed functional selectivity has even been introduced in M3 muscarinic-based designer receptors exclusively activated by designer drug (DREADD), resulting in a receptor that could engage βarrestin but could no longer activate Gq in response to the designer drug CNO^30^. However, connections between structural perturbation and signaling bias are still poorly understood. The available 3D structures of the β_2_-adrenergic receptor (β_2_AR) in both active and inactive states^10,11,21^ provide initial descriptions of receptor activation, which in combination with site-directed mutagenesis, could inform us on the structure–function basis of functional selectivity. The impact of mutations depends on the sensitivity of the site that is mutated and on the magnitude of the mutational substitution^31^. The effect of both is captured by evolutionary action (EA), an equation that models the sensitivity by the evolutionary trace (ET) method^32^, and the substitution magnitude using amino acid transition log-odds^31^. In theory, EA quantifies the evolutionary effect of genotype variations on functional responses. In practice, and spanning molecular, clinical, and population genetic observations, mutations with low EA value are mostly neutral when tested experimentally, those with high EA value drastically affect protein function, and those in between modulate function^31,33–35^. This approach generalizes prior observations that ET identifies functionally important (i.e., sensitive sequence) positions^31,36–40^. In GPCRs, mutations specifically targeted to the most evolutionary sensitive positions rewired ligand specificity of the D2 dopamine receptor by single- and multiple-site mutations^37,41^ and separated G-protein activation from βarrestin signaling by a triple-site mutation^28,42^. By considering the substitution magnitude in addition to site sensitivity, EA can now quantify the effect of specific mutations more precisely, suggesting that we may select a mutation’s site and substitution to finely tune the expected perturbation up or down.

Here, we used ET and a visual examination of the β_2_AR 3D structures to identify residues that could underlie the specific conformational rearrangements involved in the engagement of distinct signaling effectors, thereby determining the structural basis of β_2_AR functional selectivity. For the nine chosen residues, we utilized EA to select mutations intended to differentially perturb function, resulting in 28 single-site mutations. Their signaling profiles in response to isoproterenol (ISO) were characterized via cell-based assays on five different signaling pathways. Based solely on these experimental assays, non-negative matrix factorization followed by *k*-means clustering groups the mutations into three different phenotypical clusters, which are not only defined by common signaling signatures but also demonstrate shared conformational alterations, and EA scores. These observations point to multiple motifs in determining signaling specificity toward the different pathways engaged by the β_2_AR, thus providing insights in the molecular determinants of functional selectivity.

## Results

### Choice of mutations

**Fig. 1:**
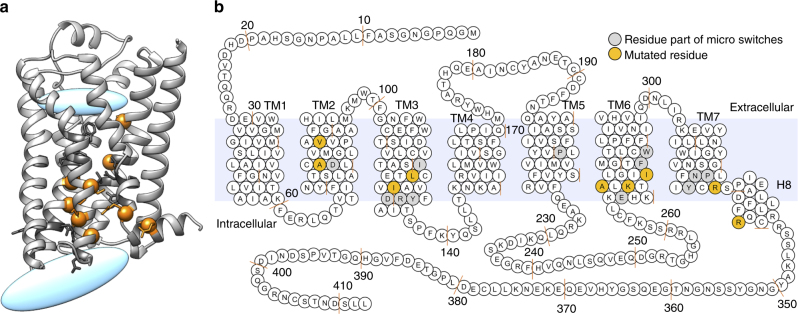
Location of mutated residues in the β_2_AR. **a** 3D representation of WT β_2_AR structure (pdb: 2RH1). Mutated residues are shown as orange spheres. Residues part of microswitches are shown in stick representation and are colored in dark gray. Blue ellipses denote the ligand binding and the effector binding regions. **b** Snakeplot of β_2_AR depicting the location of mutated residues (colored in orange). Residues part of known microswitches are colored in light gray

Amino acids to be mutated were first selected by determining the ET values of all transmembrane domain β_2_AR residues using the previously described ET method^43^. Given that intramolecular water molecules are known to play a key role in regulating protein activity^44–46^, we considered ET values, as well as their distances to the water molecules involved in hydrogen bond networks within the receptor structure to identify the residues to be mutated (Fig. 1, Supplementary Table 1). In addition, we intentionally selected residues away from the ligand binding pocket (except V86) to avoid interference with ligand binding. Accordingly, the five residues (A78^2.49^, V86^2.57^, L124^3.43^, I127^3.46^, and I278^6.40^—Ballesteros–Weinstein numbering^47^ is given in superscript) with the highest ET ranking that are located close to intra-protein water molecules and, for four of them, distant from the ligand binding pocket were selected. In order to assess the differential effects of mutation with a range of impacts on these residues^47^, we selected substitutions with a wide range of EA scores from low (<30), to moderate (30–70), and to high impact (>70, Supplementary Table 1).

In addition to the ET-based selection, visual inspection of the crystal structures of inactive and active β_2_AR lead us to also consider a cluster of positively charged amino acids (K273^6.35^, R328^7.55,^ and R333^8.51^) (Fig. 1, Supplementary Table 1) pointing outwards of the helix-bundle. The unusual orientations of these residues that could be involved in helix 8 positioning prompted us to substitute them to amino acids with distinct physicochemical properties (A→L, K→R, or R→K). Finally, A271^6.33^, a residue pointing towards the G-protein binding pocket was substituted to a bulky residue (W) or threonine (T) to assess their ability to interfere with G-protein selective coupling.

### Signaling profiles of β_2_AR variants

**Fig. 2:**
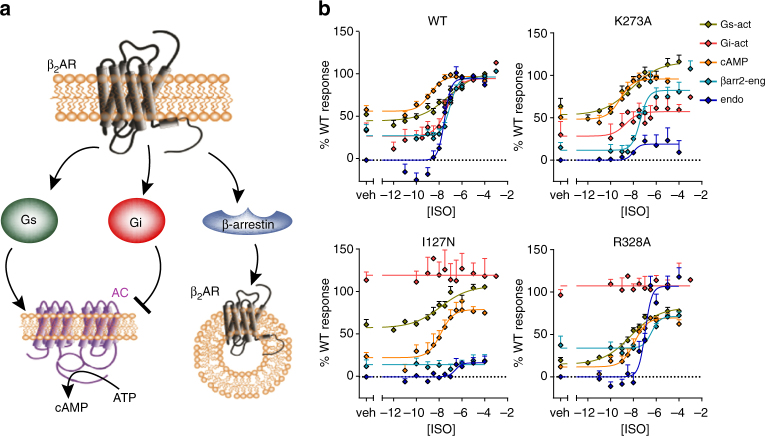
Signaling of the β_2_AR. **a** Simplified schematic of β_2_AR signaling pathways monitored in this study. **b** Concentration response curves of the five monitored pathways for WT and three variants of β_2_AR to exemplify original data. K273A is representative of mutations that yielded a signaling profile similar to WT whereas I127N exemplifies mutations increasing the constitutive activity of Gi that abrogate its ISO stimulation and reducing most of βarrestin engagement and endocytosis whereas R328A exemplifies mutations in which Gi constitutive activity is increased and ISO stimulation is abrogated but βarrestin and endocytosis are maintained. For each concentration response curve, the β_2_AR-unspecific response (value of cells only transfected with the biosensor (no receptor added)) was set to 0% and the maximal ISO-stimulated response of WT β_2_AR to 100%. The non-stimulated condition (veh) represents the basal activity in each monitored pathway. Data for all β_2_AR variants is given in Supplementary Fig. 2. For details, see Methods

In order to examine the effect of the mutations on the signaling profile of the β_2_AR, we analyzed G-protein-dependent and -independent signaling pathways. We monitored basal and ISO-induced Gs activation, Gi activation and βarrestin engagement as well as their downstream events (cAMP production and endocytosis; Fig. 2a). The responses obtained for each pathway with WT receptor are illustrated in Supplementary Fig. 1a. Concentration response curves were generated and normalized to WT β_2_AR for each pathway and β_2_AR variant. Representative examples for mutations leading to signaling signatures that are either similar to WT receptor or altered for constitutive and/or agonist-stimulated activity of specific pathways are shown in Fig. 2b. The entire data set for the 28 mutations is given in Supplementary Fig. 2. From these curves, five signaling parameters were determined by curve fitting for each pathway (Supplementary Fig. 1b, c). These parameters are basal, lSO induced and maximal activity as well as pEC_50_ and logΤ/K_A;_ except for βarrestin engagement and endocytosis, where no basal activity can be determined. Hence, the signaling profile of each receptor variant is composed of 23 parameters (Supplementary Tables 2–6).

**Fig. 3:**
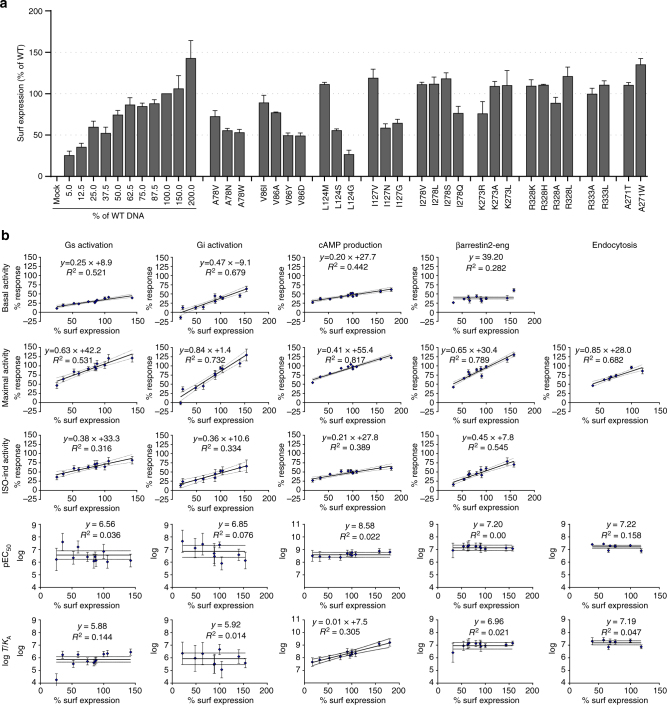
Normalizing to cell surface expression. **a** Surface expression as % of WT for WT and β_2_AR variants. The standard curve of WT β_2_AR was performed by increasing the transfected DNA amount and controlling surface expression level. Data shown are the average±s.e.m. of at least three independent experiments performed in duplicate. **b** Standard curves for all measures of WT β_2_AR were generated in order to compensate for variations in expression level. If there is a linear correlation between expression and measure, an interpolated value for wild type is used for comparison. Otherwise the variant is compared to an average WT value. For details see Methods

Mutations within TM regions of GPCRs might influence the stability of the receptor and thus the surface expression level. Therefore, we closely monitored the cell surface expression of WT and each β_2_AR variant in each experiment by cell surface ELISA (Fig. 3a). About half of the introduced mutations reduced the expression by ∼50%. Since we were unable to increase the cell surface levels of these mutated receptors, we used incremental amounts of WT β_2_AR to generate standard curves reflecting a span of expression levels encompassing the expression levels of all mutants for all signaling parameters determined (Supplementary Fig 3). Hence, we were able to compare the signaling parameters of receptor variants to interpolated parameters of WT β_2_AR at the same expression level using normalized difference (see Fig. 3b and “Methods” for details).

### Clustering of β_2_AR variants based on their signaling profiles

**Fig. 4:**
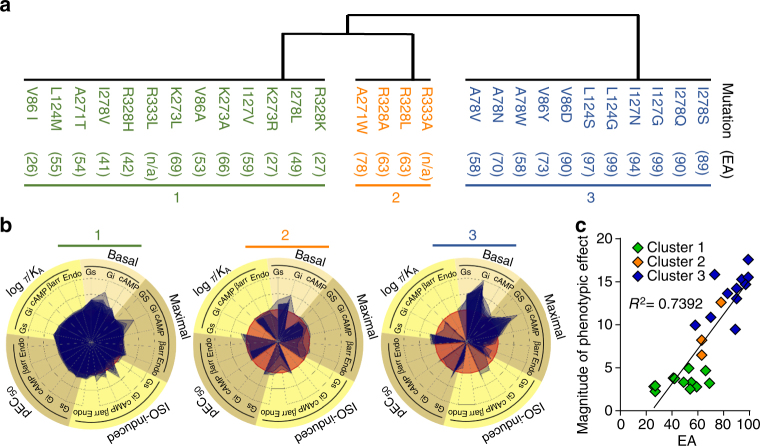
Clustering and signaling profiles of β_2_AR variants. **a** Clustering by nnmf/*k*-means method based on the signaling parameters. The clusters one to three are color coded. The EA score for each mutation is given in brackets. n/a not available. **b** Radial graphs of the signaling profile for each cluster. Each cluster is displayed by overlaying the receptor variant profiles. The data is shown as normalized difference (mut-WT divided by mut+WT). WT is shown in red as reference. Each mutant receptor is in blue, resulting in darker areas when overlayed. The signaling profiles of all receptor variants are given in Supplementary Fig. 3. **c** Correlation of EA with magnitude of phenotypic change by addition of the magnitude of change in each parameter of the signaling profile

As shown in Fig. 2b and Supplementary Fig. 2, the mutations in the β_2_AR lead to a large variety of changes in the signaling profiles. To identify commonalities among the signaling profiles, we used non-negative matrix factorization (nnmf)^48,49^ and *k*-means clustering to partition the mutations into the fewest groups within which the assay profiles were most alike and between which they were most distinct (Fig. 4a, b, Supplementary Fig. 4 and Supplementary Table 7).

The signaling profile of the largest cluster (V86I/A, L124M, I127V, A271T, K273R/A/L, I278V/L, R328K/H, R333L), cluster 1, displays only minor changes compared to WT β_2_AR. In contrast to cluster 1, cluster 2 (A271W, R328A/L, R333A) differs significantly from WT β_2_AR. Whereas Gs signaling and cAMP production were not appreciably affected, and βarrestin engagement and endocytosis were only slightly reduced, the constitutive activity toward Gi activation was considerably increased. The change in constitutive activity was accompanied by a complete loss of ISO-stimulated activity. This lack of responsiveness did not result from a loss of binding ability of the ligand since ISO-binding affinity was not affected (Supplementary Table 8) and the agonist potency towards the other pathways was not affected. Similarly, it did not result from a saturation of the assay since higher activity levels could be detected for other β_2_AR variants (ex: I278V and A271T, Supplementary Fig. 2). Similar to cluster 2, variants of cluster 3 (A78V/N/W, V86Y/D, L124S/G, I127N/G, I278Q/S) also showed increased constitutive Gi activation and loss of ISO responsiveness. The increase in basal activity was even more dramatic than that observed in cluster 2, as it reached levels that were higher than the maximal ISO-stimulated response observed for WT receptor. In addition, the ISO-stimulated βarrestin engagement and endocytosis of cluster 3 variants were dramatically reduced and their constitutive activity toward Gs activation was increased.

EA predicts the impact of mutations on phenotype. Ideally, if clusters 1, 2, and 3 accurately distinguish three phenotypes, we expect that EA scores should vary much less within each cluster than between them. Indeed, compared to a random distribution, EA scores are significantly closer within each group than between any two of them (*p* value = 1.34 × 10^−05^). As expected, cluster 1 is comprised of low, cluster 2 of medium, and cluster 3 of high EA scores. The correlation between EA scores and phenotypic mutational impact can be quantified by calculating the overall phenotypic effect of each mutation compared to wild type signaling, leading to *R* ^*2*^ value of 0.74 (*R* = 0.86, Fig. 4c).

### Structural analysis of mutated β_2_AR

**Fig. 5:**
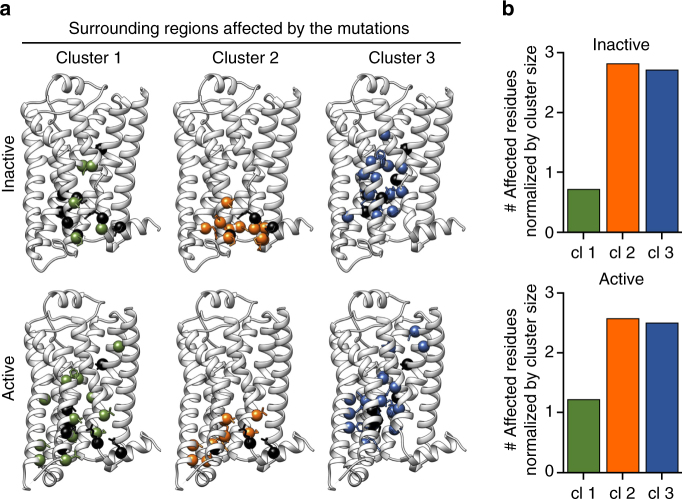
Structural changes induced by mutations in the β_2_AR. **a** Predicted changes within 4.5 Å of the mutated position of β_2_AR shown by cluster for the inactive conformation (pdb: 2RH1, top) and the active conformation (pdb: 4LDE, bottom). Residues are colored by cluster (mutated residues—black, cluster 1—green, cluster 2—orange, cluster 3—blue) and depicted as spheres. **b** Number of affected residues in the inactive (top) and active (bottom) conformation normalized by cluster size

We used in silico mutagenesis and energy minimization to predict the structural changes induced by the mutations. For this purpose, the changes in the neighborhood of each mutation (residues entering and exiting a 4.5 Å radius around the mutation site) were predicted using the Molecular Operating Environment (MOE) structure-based design package^50^. The affected residues were then grouped according to the phenotypical clustering of the mutations, and mapped on the inactive and active X-ray structures of the β_2_AR (Fig. 5a). The positions of the residues predicted to be affected for each of the individual mutations are illustrated for both the inactive (Supplementary Fig. 5) and active (Supplementary Fig. 6) receptor conformations.

The perturbations promoted by mutations of cluster 1 were dispersed throughout the receptor for both the inactive and the active structure without any appearance of structural grouping. The average number of residues affected by each mutation was smaller in cluster 1 than either cluster 2 or 3 (Fig. 5b), consistent with the modest functional consequences observed for these mutations. The affected residues by the cluster 2 mutations grouped in the lower third of the receptor’s TM domain, whereas cluster 3 mutations and resulting perturbations were mainly found around the middle of the receptor’s TM domain.

**Fig. 6:**
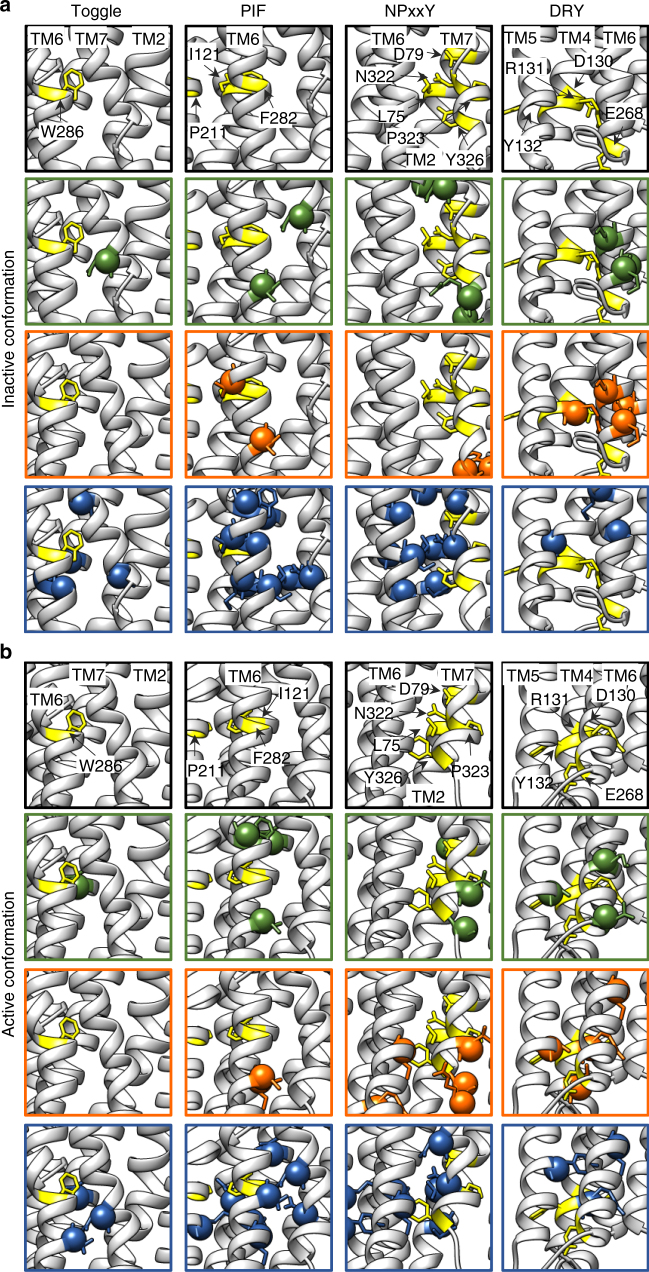
Influence on microswitches. Predicted changes in the environment of four microswitches (toggle-W286^6.48^, PIF-P211^5.50^, I121^3.40^, F282^6.44^, NPxxY-N322^7.49^,P323^7.50^,Y326^7.53^,L75^2.46^,D79^2.50^, DRY-D130^3.49^,R131^3.50^,Y132^3.51^,E268^6.30^) in **a** the inactive (pdb: 2RH1) and **b** the active conformation (pdb: 4LDE). The predicted changes are colored by cluster (cluster 1—green, cluster 2—orange, cluster 3—blue) and depicted as spheres. Residues of the microswitches and known interaction partners are colored yellow, shown in stick representation and labeled in the upper most row of **a** and **b**

The activation process of GPCRs is conveyed by conformational rearrangements of key residues in microswitches. Hence, we investigated whether the predicted clustered changes in cluster 2 and 3 neighbor known microswitches (PIF motif/connector, DRY motif, NPxxY motif, Toggle switch). For cluster 2, the majority of the changes centered around the G-protein binding site, which is flanked by the DRY and NPxxY motifs. The alterations in cluster 3 occur both around the PIF motif as well as the NPxxY motif (Fig. 6).

The prediction of structural changes is consistent with the phenotypic clustering since mutations in the different clusters resulted in distinct structural perturbations. Receptor variants in cluster 2 that displayed increased constitutive Gi activity and loss of ISO-induced Gi activation are characterized mainly by changes around the G-protein binding site (DRY/NPxxY). For cluster 3 variants, which showed increased basal activity for both G-proteins and also lost ISO-promoted Gi activation and βarrestin engagement, mutations were predicted to affect the PIF and toggle switches in addition to the NPxxY, consistent with a greater functional impact.

These observations suggest that different regions of the receptor may be important for the stabilization of different receptor conformations causing preferential signaling through various effectors.

## Discussion

GPCR signaling is pleiotropic, and while the basis of GPCR functional selectivity is not fully characterized, perturbations to the structural conformation have been implicated in biased signaling^13,24,25,39,51^. To better characterize the structure–function relationship in GPCR signaling bias, we combined complementary approaches including: computational predictions via ET and EA analyses, experimental receptor signaling profiling via BRET-based biosensors and molecular modeling. First, ET and EA were combined to select and tune the intensity of mutational perturbations at sequence positions that are themselves predicted to be more or less functionally sensitive. Second, in order to disentangle the heterogeneous signaling outputs typical of GPCR signaling, we utilized BRET biosensors to characterize the diversity of these pathways. Third, computational comparison, through nnmf and clustering analyses, grouped the complexity of each mutation’s response profile into a simpler and robust classification of the mutation’s global effect on the entirety of receptor signaling profile rather than being limited to a specific pathway-based analysis. Taken together, these steps therefore form a comprehensive computational and experimental pipeline to predict, test and validate mutationally induced receptor bias allowing targeted site-specific mutagenesis and reengineering of receptor function. While we have demonstrated this approach within the β_2_AR, all the methodologies used were designed for application in any protein and therefore represent a high throughput methodology to interrogate structure–function relationships.

In this study, we demonstrated the pipeline’s efficacy to characterize perturbations to the β_2_AR signaling profile using 28 single-site mutations at nine different structural positions important to receptor function. These 28 mutations clustered into three different major phenotypic outcomes: (i) minimal perturbation compared to WT signaling, (ii) increased basal activity for Gi activation together with loss of ligand-induced Gi activation, and (iii) increased basal activity for G-protein activation and loss of both ligand-induced Gi and βarrestin responses. Noticeably, only a few mutations affected agonist-stimulated Gs activity so that it did not contribute to the clustering (Supplementary Fig. 4).

Strikingly, we note a highly significant trend between predicted impact by EA and actual phenotypic perturbation (Fig. 4c), as previously shown in a retrospective analysis^31^. EA is a first principle equation for evolution. It uses calculus, the mathematical language for the study of variations, to formally link genotype variations, or mutations, to their effect on phenotype or functional readouts. Our results show that even in a multi-functional system as complex as GPCRs, the EA score accurately quantifies the impact of coding mutations on the loss of downstream signaling. EA predictions are validated both on the broad scale, capturing the overall phenotypic change and correlating with the cluster assignments, and on the small scale, as individual mutations at the same structural position result in different signaling biases depending on the severity of the mutation. This trend is exemplified by V86I (EA score of 26), V86A (53), V86Y (73), and V86D (90) (Supplementary Fig. 4) where we see a gradual increase in experimental signaling perturbation as a function of increasing EA scores, substantiating the notion that different receptor conformations engage different effector proteins. We do however note that mutations within cluster 1 deviate from the linear correlation, with some EA predictions being higher than their measured phenotypic response. This is most likely due to the fact that the lower impact of these mutations cannot be detected experimentally by the assays used. We can speculate that either the assays were not sufficiently sensitive to detect the signaling impact or that these mutations affect a signaling pathway that was not assessed in the present study.

Mutations predicted to have a low impact all caused minimal modulation of β_2_AR signaling and clustered together in cluster 1. The weakness of this biological perturbation is consistent with the relatively minor structural alterations to the β_2_AR structure inferred from structural modeling. Thus, a native-like structural conformation exhibits wild-type-like signaling.

Medium impact mutations in cluster 2, as expected, show moderate signaling changes correlating to structural perturbations mainly affecting the Gi pathway, both in the basal and the ISO-induced activity. In agreement with previous findings on EA, these mid-range mutations (EA around 50) only perturb specific aspects of protein function rather than have a universally deleterious effect. Interestingly, the predicted structural changes for these mutations are centered around the active conformation of the G-protein binding site, including the NPxxY and DRY motif. While the R131^3.50^ of the DRY motif has been implicated in direct binding of the G-protein^11,14^, the shape of the G-protein binding pocket itself also plays a role in G-protein binding and potentially in G-protein selectivity. Recently, it has been suggested that the position of TM6 determines G-protein specificity. A smaller G-protein binding pocket would favor Gi binding due to its slimmer C-terminus, whereas a larger G-protein binding pocket favors Gs binding^52^. Given their impact around both the DRY motif and the G-protein binding pocket, the predicted alterations induced by the mutations in cluster 2 might allow TM6 an easier transition towards the Gi active state thereby explaining increased basal activity. Furthermore, the fact that no increase in Gi activation can be induced by ISO indicates that the fraction of receptors in active state is ligand independent, suggesting a mutation-induced receptor conformation that is uncoupled from the “normal” allosteric regulation of the Gi pathway during receptor activation.

High impact mutations in cluster 3 share a signaling bias against agonist-promoted Gi- and βarrestin-signaling and an increased basal Gs and Gi activity. Cluster 3 mutations are predicted to promote similar alterations in the active state G-protein binding site (albeit more centered around the NPxxY than the DRY motif) as cluster 2, which likely contributes to the increased basal activity and reduced ISO-induced activity towards Gi activation. However, the drastically increased constitutive Gi and Gs activity and loss of agonist-promoted βarrestin engagement suggest other causes. Across all mutations in cluster 3, we note a significantly altered structural conformations around the PIF and NPxxY motifs, which are not present in cluster 1 or 2. Structural rearrangements in the PIF motif during activation have been described for various receptors (β_2_AR^10,23^, 5HT^13^, μOR^46^). So far, these rearrangements have been loosely associated with G-protein signaling^13^, however, no in depth functional analysis on this motif has been performed. The NPxxY motif in TM7 was shown to be important for normal receptor function, including G-protein signaling and receptor internalization^53,54^. Furthermore, the conformation of TM7 was linked to βarrestin engagement^13,24,55^. Our results are in agreement with these analyses and implicate both the PIF and NPxxY motifs in disruption of receptor signaling, constitutively active G-protein activation, and the elimination of βarrestin signaling.

Within cluster 3, some mutations exclusively affect the NPxxY motif while others only affect the conformation of the PIF motif (Supplementary Figs. 5 and 6), yet all mutations within this cluster have a conserved phenotype in regards to the Gi and βarrestin pathway. These data suggest that alterations in either motif are sufficient to cause the observed biased signaling profile. Given the nature of the PIF motif to transduce signal from the ligand binding event through the TM region, we cannot exclude NPxxY involvement in mutations that only affect the PIF motif. It is possible that the alterations of the PIF motif result in an identical phenotype to alterations directly to the NPxxY motif due to a coupling of PIF to the NPxxY motif. An alternative explanation is that disruptions to the PIF motif cause phenotypic alterations independent of the NPxxY motif, indicating that different receptor conformations might have the same signaling output. Another possibility is that the PIF and the NPxxY motifs act in parallel and that both rearrangements are needed for some of the signaling activity. Hence, alterations to either one of these two microswitches could result in the same functional outcome. Regardless, our data, in agreement with prior research, implicates both the PIF domain and the NPxxY motif in receptor activation and signaling through Gi and βarrestin. Additionally, these data suggest a novel function of the PIF motif in modulating the activation of βarrestin signaling in concert with the NPxxY motif.

These results highlight the power of EA to inform future mutagenesis and receptor engineering by quantifying the expected impact a mutation has on protein function. Designed mutations can therefore potentially be fine-tuned to the desired impact, thereby reducing time spent on random mutagenesis to achieve a desired function. This is illustrated by the fact that when transfering three substitutions (I to V, L or Q) at position I^6.40^ from the β_2_AR (I278) to the vasopressin type 2 receptor (V2R) (I276), we found that, as was the case for the β_2_AR, the I276V/L variants had no or modest effects on either Gs activation, cAMP production or βarrestin recruitment whereas I276Q considerably and selectively affected βarrestin recruitment (Supplementary Fig. 7).

The approach followed in the present study has obvious implications for a possible understanding of the structural determinants of ligand-biased signaling. Further studies assessing the effects of mutations selectively affecting Gi or βarrestin engagement on the activity of βarrestin- or G-protein-biased ligands should shed new light on the specific and/or local conformational rearrangements required to engage specific signaling effectors. Structural studies have already highlighted that ligands with distinct functional selectivity profiles result in distinct receptor conformation^26,56,57^. Based on NMR signals, Liu et al.^24^ reported that β-arrestin-biased ligands predominantly impact the conformational states of TM7. Interestingly, mutations selectively affecting βarrestin engagement (cluster 3) are predicted to have more impact on residues close to the PIF and NPXXY than the DRY motif. Assessing how these mutations may affect the movement of TM7 upon binding of balanced and biased ligands will be of considerable interest.

In summary, we have developed a comprehensive methodology capable of guiding site-directed mutagenesis studies and protein reengineering by coupling in silico evolutionary lineage analysis with biological characterization and collaborative filtering clustering techniques. Using this approach, we demonstrate a strong correlation between our computational predictions stemming from evolutionary history, the actual biological perturbations and structural determinants thereof. Additionally, EA enables the design of mutations at the same position, which vary gradually in functional effect and magnitude, in effect tuning them like a turn of a rheostat. Even though our designed mutations did not occur directly within the functional motifs, the resulting structural changes altered key functional microswitches, further demonstrating the power of this approach to arrive at non-obvious solutions to signaling perturbation. Through this validation, we have gained a better understanding of the molecular determinants of biased GPCR conformations, a crucial first step for the development of biased ligands that activate only beneficial pathways thereby reducing pharmacological side effects and predicting how clinically relevant mutations result in altered cellular signaling leading to disease progression.

## Methods

### Reagents

(-)-Isoproterenol hydrochloride (ISO) was purchased from Sigma Aldrich (St. Louis, MO, USA). Coelenterazine 400a was purchased from NanoLight Technology (Pinetop, AZ, USA). All cell culture reagents were bought from Wisent (St-Bruno, QC, Canada).

### Plasmids

Single-site mutations in the β_2_AR and V2R were introduced via PCR with QuickChange Site-directed Mutagenesis Kit (Agilent Technologies, Santa Clare, CA, USA) following the manufacturer’s instructions. All β_2_AR variants were verified by sequencing. Receptor constructs contained an Ha tag at their N-terminus, as previously described^58,59^, to facilitate the quantification of cell surface expression by ELISA.

For creating a unimolecular biosensor for monitoring βarrestin recruitment, GFP_10_ coding sequence (cds) without stop codon was PCR amplified with a forward primer encoding the plasma-membrane targeting sequence from the Lyn Kinase and a small linker (MGCIKSKGKDSLSNA), RlucII cds without stop codon was PCR amplified and both fragments assembled in pCDNA 3.1 Zeo(+) using In-Fusion (Clontech Laboratories, Mountain View, CA, USA), creating pCDNA3.1 Zeo(+) Lyn-GFP_10_-RlucII. The cds of a structurally disorganized 300 residues-long linker, previously described (Dis300LNK)^60^, was subcloned in between GFP_10_ and RlucII, creating pCDNA3.1 Zeo(+) Lyn-GFP_10_-Dis300LNK-RlucII. The cds of βarrestin2 was PCR amplified using forward primers encoding a small flexible linker (GSGSAGTA) and inserted at the C-terminus of RlucII, creating pCDNA3.1 Zeo(+)βarr2 trans constructs.

### Cells, cell culture, and transfections

BCM3 is a clone of the HEK293T cell line in which BRET-based biosensors have been developed in Dr Bouvier’s laboratory and was used for all the BRET and ELISA experiments. Cells were regularly tested for mycoplasma contamination (PCR Mycoplasma Detection kit, abm, BC, Canada) and only mycoplasma-negative cells were used for the assays.

HEK293T cells were grown at 37 °C with 5% CO_2_ in Dulbecco’s modified Eagle’s medium (DMEM) supplemented with 10% fetal bovine serum. HEK293T cells were transiently transfected (500,000 cells per well) in six-well plates with biosensors for bioluminescence resonance energy transfer (BRET) assays and ELISA with X-treme GENE 9 DNA Transfection Reagent (Transfection Reagent:DNA ratio: 3:1; Roche Diagnostics, Indianapolis, IN, USA). Cells were re-plated (50,000 cells per well) 24 h post transfection into white 96 well Culture Plates (Greiner Bio-One GmbH, Frickenhausen, Germany) coated with poly-l-ornithine. The day of the experiment, cells were washed twice with stimulation buffer (Modified Hank’s Balances Salt Solution (HBSS): 137 mM NaCl, 5.4 mM KCl, 0.25 mM Na_2_HPO_4_, 0.44 mM KH_2_PO_4_, 1.8 mM CaCl_2_, 0.8 mM MgSO_4_, 4.2 mM NaHCO_3_, 0.2% (w/v) d-glucose, pH 7.4).

The activity of each signaling pathway was measured in living cells using BRET-based assays described below. Optimal times of measurement following agonist stimulation were determined from time-course experiments carried out for each assay for the WT receptor. Times for which maximal responses was achieved and stable for a given assay were selected and were 5, 15 and 30 min for G-protein activity, βarrestin recruitment and cAMP production assays, respectively. The ELISA-based endocytosis assay was carried out 60 min following agonist stimulation.

### Gs- and Gi-activation assay

HEK293T cells were co-transfected with varying amounts of WT or β_2_AR variants (250 ng per well) and a three-component G-protein activation biosensor composed of Gα-RlucII (Gαs-67-RlucII (5 ng per well)^61^ for Gs activation or Gαi_2_-99-RlucII (1 ng per well)^62^ for Gi activation), Gβ1 (100 ng per well), and GFP_10_-Gγ1 (25 ng per well) as described above. Coelenterazine 400a, diluted in stimulation buffer (final: 2.5 µM) was added to the wells for 6 min, then ISO, diluted in stimulation buffer, was added at the indicated concentrations to the wells for 5 min. Plates were read on the Mithras LB 940 (Berthold Technologies, Bad Wildbad, Germany), for 1 s per well, with filters set at 410 ± 70 nm (RlucII) and 515 ± 20 nm (GFP_10_) and BRET ratios were calculated as GFP_10_ divided by RlucII. Upon formation of the Gα-RlucII:Gβ1:GFP_10_-Gγ1 hetero-trimer, a significant BRET signal is detected resulting from the proximity between Gα-RlucII and GFP_10_-Gγ1. Upon activation of Gα, the separation between the Gα and Gβγ subunits leads to a decrease in BRET^61^.

### cAMP production assay

HEK293T cells were co-transfected with varying amounts of WT or β_2_AR variants (200 ng per well) and BRET-cAMP biosensor (GFP_10_-mutEPAC1-RlucII; 1 ng per well)^63^. The indicated concentrations of ISO, diluted in stimulation buffer, were added to the wells for 30 min, then coelenterazine 400a, diluted in stimulation buffer (final: 2.5 µM) was added to the wells for 5 min. Plates were read on the Mithras LB 940, as described above. Upon binding of cAMP, EPAC undergoes a conformational change leading to a significant decrease in the observed BRET signal.

### βarrestin2 recruitment

HEK293T cells were co-transfected with varying amounts of WT or β_2_AR variants (250 ng per well) and the BRET-βarrestin2-recruitment biosensor (βarr2 trans constructs, 2 ng per well). The indicated concentrations of ISO, diluted in stimulation buffer, were added to the wells for 15 min, then coelenterazine 400a, diluted in stimulation buffer (final: 2.5 µM) was added to the wells for 5 min. Plates were read on the Mithras LB 940, as described above. Upon recruitment of βarrestin2 to the receptor, a significant increase in BRET signal is observed resulting from the increased proximity between RlucII and GFP_10_ upon translocation of the βarrestin to the stimulated receptor.

### Endocytosis

HEK293T cells were transfected with WT or β_2_AR variants (50 ng per well) as described above. The indicated concentrations of ISO, diluted in stimulation buffer, were added to the wells for 60 min, then a cell surface ELISA was performed. In short, cells were fixed with 3% paraformaldehyde for 10 min. Cells were washed three times in washing buffer (modified HBSS (see above) +0.5% BSA), then cells were incubated for 60 min with anti-HA-peroxidase antibody (12013819001, Roche Diagnostics, Indianapolis, IN, USA) (1:1000 in washing buffer). Cells were washed three times in washing buffer. ECL (1:1 ratio; Perkin Elmer, Waltham, MA, USA) was added to the wells and plates were read on the Mithras LB 940, for 1 s per well, with no filters.

### Cell surface expression

In order to monitor cell surface expression of WT or variants β_2_AR transfected cells used for BRET or endocytosis assays, cell surface ELISA was performed on cells re-plated 24 h post transfection, as for endocytosis experiments but without ISO stimulation.

### Analysis of concentration response curves

In order to correct for β_2_AR-unspecific response, the value of cells only transfected with the biosensor (no receptor added) was subtracted from the experimental value. For each repeat of the assays, data were normalized as percentage of WT β_2_AR maximal ISO-stimulated response (Supplementary Fig. 1b, c). Signaling parameters: basal activity, maximal activity, ISO-induced activity, and pEC_50_ were determined by fitting the concentration response curve to Eq. (1):

1 $${{E = }}\:{\rm {basal}} + \frac{{{\rm {max}} - {\rm {basal}}}}{{1 + 10^{({\rm {logEC}}_{50} - [A])*{{n}}}}},$$

where *E* is the effect of the ligand, [*A*] is the concentration of the ligand, max is the maximal response, basal is the non-stimulated response, and *n* is the slope of the transducer function that links occupancy to response.

The signaling parameter log *Τ*/*K*_*A*_ was determined by fitting the concentration response curve to Eq. (2):

2 $$E\;{\mathrm{ = basal}} + \frac{{{\mathrm{max}} - {\mathrm{basal}}}}{{1 + \left( {\frac{{\frac{{10^{[A]}}}{{10^{{\mathrm{log}}K_A}}} + 1}}{{10^{{\mathrm{log}}R} \times 10^{[A]}}}} \right)^n}},$$

where *E* is the effect of the ligand, [*A*] is the concentration of the ligand, max is the maximal response, basal is the non-stimulated response, log*K*_*A*_ denote the logarithmic functional equilibrium dissociation constant of the ligand, *n* is the slope of the transducer function that links occupancy to response, and log*R* is the logarithm of the “transduction coefficient”, *Τ*/*K*_*A*_, where *Τ* is an index of the coupling efficiency of the agonist. For detail see refs. ^64,65^

In Eqs. (1) and (2), *n* was fixed to the determined value for WT β_2_AR and basal was not fixed. In Eq. (2) max was shared for all data sets. Values of *n* for WT β_2_AR used in the fitting are given in Supplementary Fig. 1.

### Correction for cell surface expression

For each assay, a concentration response curve for WT β_2_AR using 5–150% of DNA amount were prepared and the cell surface expression was measured. The signaling parameters (basal activity, maximal activity, ISO-induced activity, pEC_50_, and log *Τ*/*K*_*A*_) were determined and their correlation to the expression level was calculated (Fig. 3b). If *R* ^2^ was higher than 0.3, a linear correlation was presumed and a “theoretical WT value” with the surface expression level of each mutant was calculated using the determined equation. If *R* ^2^ was lower than 0.3, the average of all values was used as “theoretical WT value”. In order to compare the signaling parameters of β_2_AR variants to WT β_2_AR, Eq. (3) was used:

3 $$\Delta _{{\mathrm{norm}}} = \frac{{{\mathrm{mut}} - {\mathrm{WT}}_{{\mathrm{theoretical}}}}}{{{\mathrm{mut}} + {\mathrm{WT}}_{{\mathrm{theoretical}}}}},$$

where Δ_norm_ is the normalized difference, mut denotes the value of the β_2_AR variant, and WT_theoretical_ corresponds to the interpolated value obtained from the WT receptor titration curves obtained for each pathway (Supplementary Fig. 3, Fig. 3). This normalization, which yields values between −1 and +1 (WT being 0 by definition) was done in order to allow direct visual comparison between the parameters without having different scales. Δ_norm_ is then plotted for each parameter as the radius of the radial graph.

### Evolutionary trace analysis

To identify functional residues, ET takes a set of homologs as input. Due to the variation in the GPCR loop regions, we focused solely on the transmembrane domains, a total of 195 residues. The multiple sequence alignment of the transmembrane region was made up of 2512 Class A GPCRs, excluding olfactory receptors, and constructed as previously described^37^. The sequences were gathered from the GPCRdb database (http://gpcrdb.org/) and included sequences from mammals, amphibians, reptiles, fish, birds, echinoderms, and protostomes. To select key functional amino acids, the ET results were projected on to the protein structure for β_2_AR (pdb: 2RH1). Targets were chosen based on their ET scores, their distance to the ligand and to the intramolecular waters.

### Evolutionary action scores

EA represents a first principle equation describing the fundamental basis for evolution by providing an evaluable Eq. (4) connecting the changes in genotype to their phenotypic effect:

4 $${\mathrm{d}}\varphi = \nabla f\;{\mathrm{d}}\gamma ,$$

Formally the change in phenotype (d*φ*) is equal to the evolutionary fitness gradient at that position (d*γ*), calculated by ET, multiplied by the magnitude of the substitution made, calculated by the log odds of the substitution ($\nabla f$)^31^, which is dependent on the ET score of that position and any available secondary structural information. A unique set of log odds is therefore calculated for every permutation of ET score bins (e.g., EA=1–10 or EA=10–20) with the available secondary structure information (e.g., helix, beta-sheet or coil). When structure is not available, a purely sequence-based EA score is used.

### Robustness of the clustering method

In order to establish robustness of the clusters, we propagated random experimental error through the clustering procedure. To accomplish this, we generated 1000 matrices (28 mutation×29 phenotypes, 5 pathways×6 parameters (basal, max, ligand induced, EC_50_, log *T*/*K*_*a*_, with no basal for barr) by randomly sampling, for each phenotype data point, a single value from the normal distribution with the mean and standard deviation estimated from the mutation replicates. Each point was then normalized using the normalized difference detailed above. We then performed the clustering method, detailed below, independently on each of the 1000 sampled matrices. Finally, we quantified how frequently each mutation clustered together in each of these 1000 runs, which resulted in a clustering frequency matrix (28 mutations×28 mutations). To obtain final cluster assignments and create the similarity dendrogram, the frequency matrix was converted to a distance matrix using Pearson’s correlation as a measure of similarity with final cluster designation assigned via hierarchical clustering.

For each independent run using a corresponding sampled matrix, we utilized multiple iterations of non-negative matrix factorization (nnmf)^48,49^ to cluster β_2_AR variants based on their signaling signature and their expression. Prior to factorization, values were normalized within each column (assay phenotype measurement) on a scale of 0–1 using Eq. (5)

5 $${\mathrm{normalization}} = \frac{{{\mathrm{mut}} - {\mathrm{min}}}}{{{\mathrm{max}} - {\mathrm{min}}}}.$$

where mut denotes the value of the β_2_AR variant and max and min represent the maximal and minimal values for each column. This normalization was necessary to prevent differences in phenotype scale from biasing the feature reduction step. The resulting mutant×phenotype was deconstructed into its basis vectors [*W*,*H*] (where *W* has the dimensions 28 mutations by *k*, and *H* has the dimension *k* by 28 signaling parameters and expression levels) using the multiplicative algorithm of nnmf with 500 replicates. Cluster assignment was performed using *k*-means on the *W* basis vector where *k* equaled the number of features used by nnmf. This methodology was then repeated 100 times to measure how frequently any two mutations clustered together, resulting in a mutation by mutation frequency matrix with each value being the frequency that two mutants co-clustered (Supplementary Table 7). This frequency provides a similarity measure between any two mutants.

Cluster assignment for each of the 1000 sampled matrices was conducted independently using the Pearson’s correlation method. This cluster assignment for each sampled matrix was then used for the final clustering frequency matrix, detailed above.

We performed this analysis with *k* = 2 up to *k*=7 to identify the optimal number of clusters to accurately describe the data. The intermediate frequency matrix enabled a measure to quantify clustering robustness. Specifically, cluster assignments with frequencies equal to 0% and 100% indicate very robust and distinct assignments as mutations do not jump around between multiple clusters.

### EA score clustering

For each *k*, we performed 10,000 random simulations permuting the EA scores for all mutations and measuring the variation between EA score in each cluster, where variation=EA_*i*_-EA_*j*_ for all combinations of EA scores within that cluster. All of these values were appended into an array and compared to the phenotypic clusters using a Kolmogorov–Smirnov test. This process was repeated 10,000 times and the average *P* value was used to determine significance.

### Structure prediction and analysis

Protein structure prediction of the mutated receptors was performed using the MOE structure-based design package^50^. First for both active and inactive receptor templates (2RH1 and 4LDE), the automated structure preparation protocol Protonate3D^66^ was run. Protonate3D calculates the optimal protonation states, including titration, rotamer and “flips” using a large-scale combinatorial search. Using Residue Scanning in the Protein Design panel, the intended mutations were inserted. The selection of side chain conformations is made from a rich rotamer library followed by a refinement protocol based on force field energy minimization. For this purpose, the AMBER12EHT force field was used.

Structural predictions were then visualized using the chimera visualization system^67^. Residues within 4.5 Å of the mutations sites were identified via chimera both in WT and mutated receptors. By comparing these environments, changes in their amino acid composition (both entering and existing amino acids within this 4.5 Å radius) were detected. Additionally, it was analyzed with chimera which of these changes appeared within 4.5 Å of known microswitches. Detected changes were back projected on the 3D structure and highlighted as indicated in the figure legends.

### Data analysis

Data analysis was performed using Microsoft Excel (Microsoft, Redmond, WA, USA), GraphPad Prism (versions 6, GraphPad Software, La Jolla, CA, USA) MATLAB and Statistics Toolbox Release 2014b (The MathWorks, Inc. Natick, MA, USA), and NumPy and SciPy packages^68^.

### BRET-based binding assay

Nluc (Promega, Madison, WI, USA) was inserted N-terminally into WT β_2_AR using the Gibson Assembly Kit (NEB, Ipswich, MA, USA) according to manufacturer’s instruction. Both domains were joined by an eight amino acid Gly-Ser-linker. In order to improve expression, the reported export signal^69^ was added to the construct. The signaling and binding properties of the construct were verified (Supplementary Fig. 8). Single-site mutations in the β_2_AR were introduced as described above.

HEK293T cells were transfected with Nluc-β_2_AR WT or variants (100 ng per well) as described above. Cells were re-plated (50,000 cells per well) 24 h post transfection into white 96 well cellGrade Plates (Brand GmbH und Co KG, Wertheim, Germany) coated with poly-l-ornithine. Cells were washed twice with stimulation buffer (Modified HBSS: mentioned above). 100 nM (S)-propranololol-green (labeled with BODIPY-FL; CellAura, Winscombe, UK) and increasing concentrations of ISO, diluted in stimulation buffer, were added to the wells for 30 min, then coelenterazine 400a, diluted in stimulation buffer (final: 0.1 µM) was added to the wells directly before reading. Plates were read on the GloMax® 96 Microplate Luminometer (Promega, Madison, WI, USA), for 0.5 s per well, with filters set at 465 ± 25 nm (Nluc) and 530 longpass (BODIPY-FL). BRET ratios were calculated as BODIPY-FL divided by Nluc. Upon binding of the fluorescent ligand to the Nluc-tagged receptor, a significant BRET signal is detected. The non-labeled ligand displaces the fluorescent ligand leading to a decrease in the BRET signal. The binding affinity of ISO was determined from the IC_50_s of the ISO-promoted decrease in propranolol-green binding for each variant.

### Data availability

The authors declare that all data supporting the findings in this study are presented within the article and its Supplementary Information Files, and are available from the corresponding author upon request.

## Electronic supplementary material

Supplementary Information
