# Supplementary material for: Evolutionary action and structural basis of the allosteric switch controlling β2AR functional selectivity
Source: Nat Commun. 2017 Dec 18;8:2169. doi: 10.1038/s41467-017-02257-x (PMC5735088; doi:10.1038/s41467-017-02257-x)
Supplement: Supplementary file 1 — Supplementary Information [file 41467_2017_2257_MOESM1_ESM.pdf]

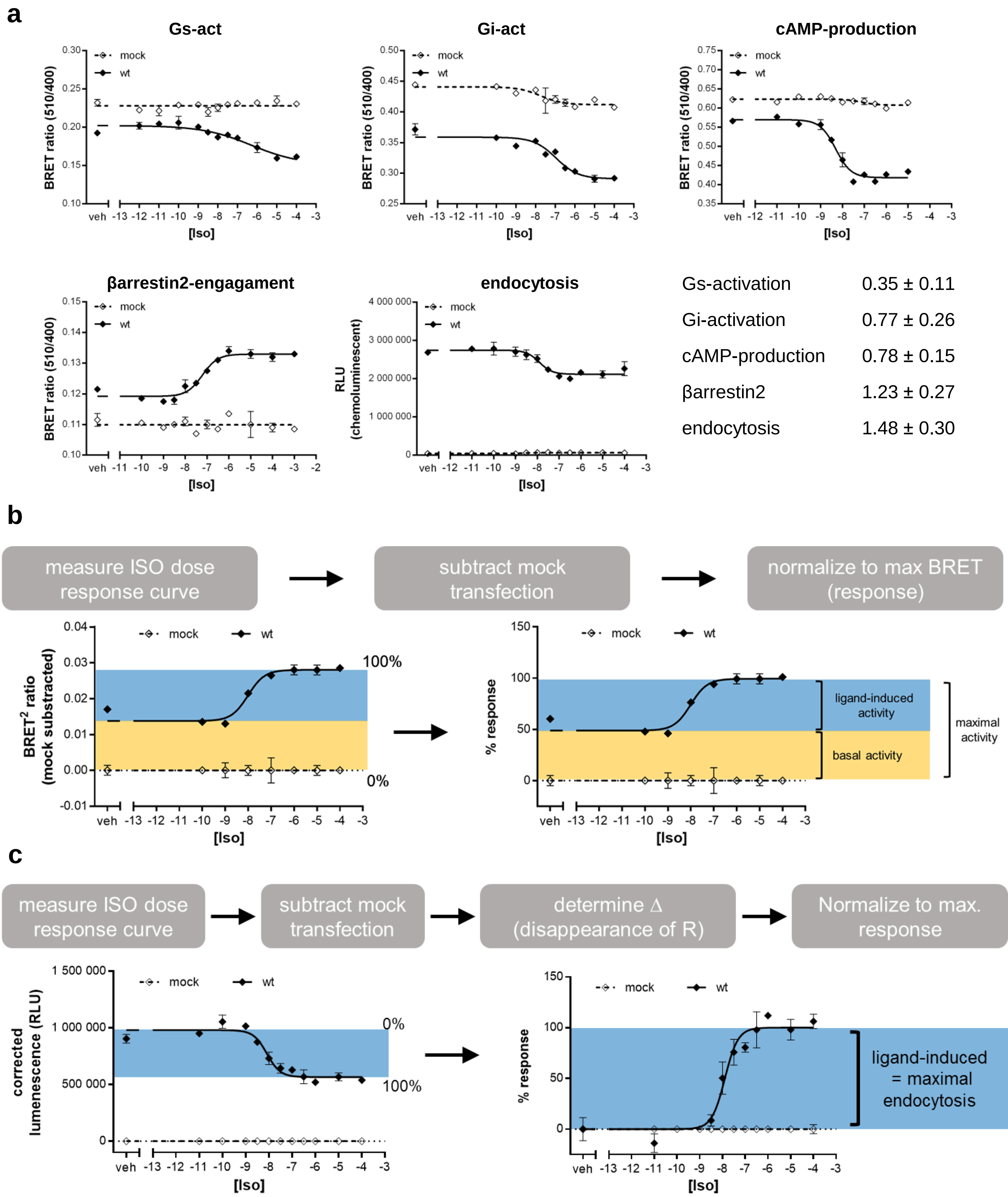

Supplementary Figure 1 | β$_2$AR data analysis. (**a**) Raw data of concentration response curves of β$_2$AR for the five monitored pathways were determined with log(agonist) vs response -- Variable slope (four parameters) in Prism. Fitted values for the Hill slope were also used when fitting the β$_2$AR variants and are given here. Data shown are representatives of at least 3 independent experiments performed in duplicate. Data analysis (**b**) for BRET-based assays and (**c**) ELISA-based assay. For details see Methods.

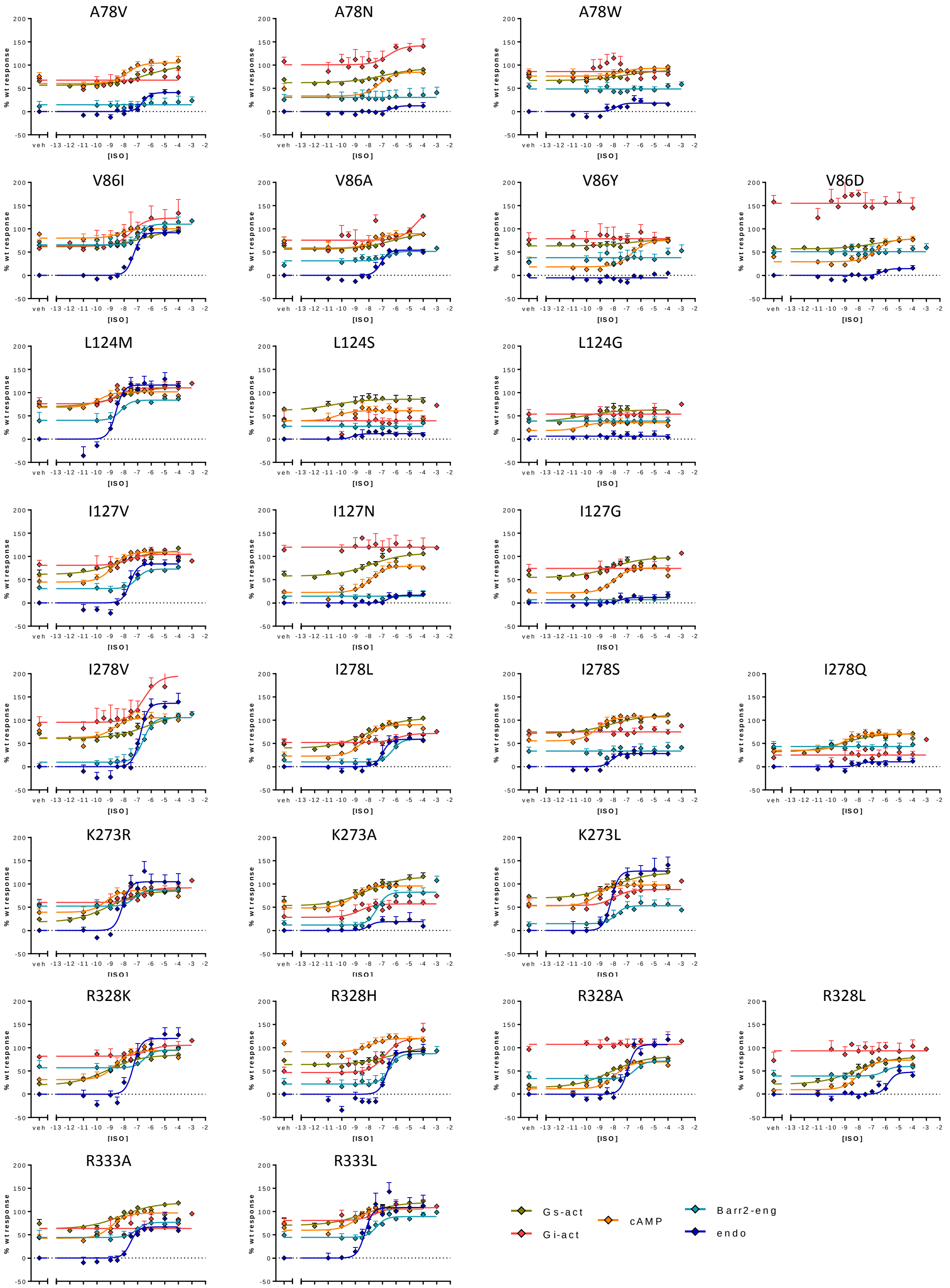

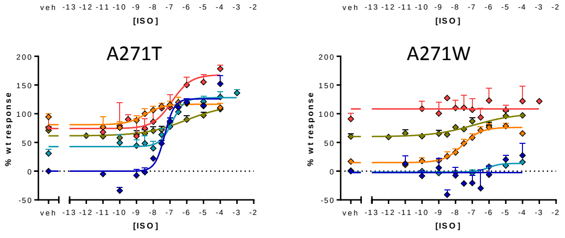

Supplementary Figure 2 | Experimental results. Concentration response curves for five monitored pathways of 28 β_2AR variants. Data shown are the average ±s.e.m. of at least 3 independent experiments performed in duplicate.

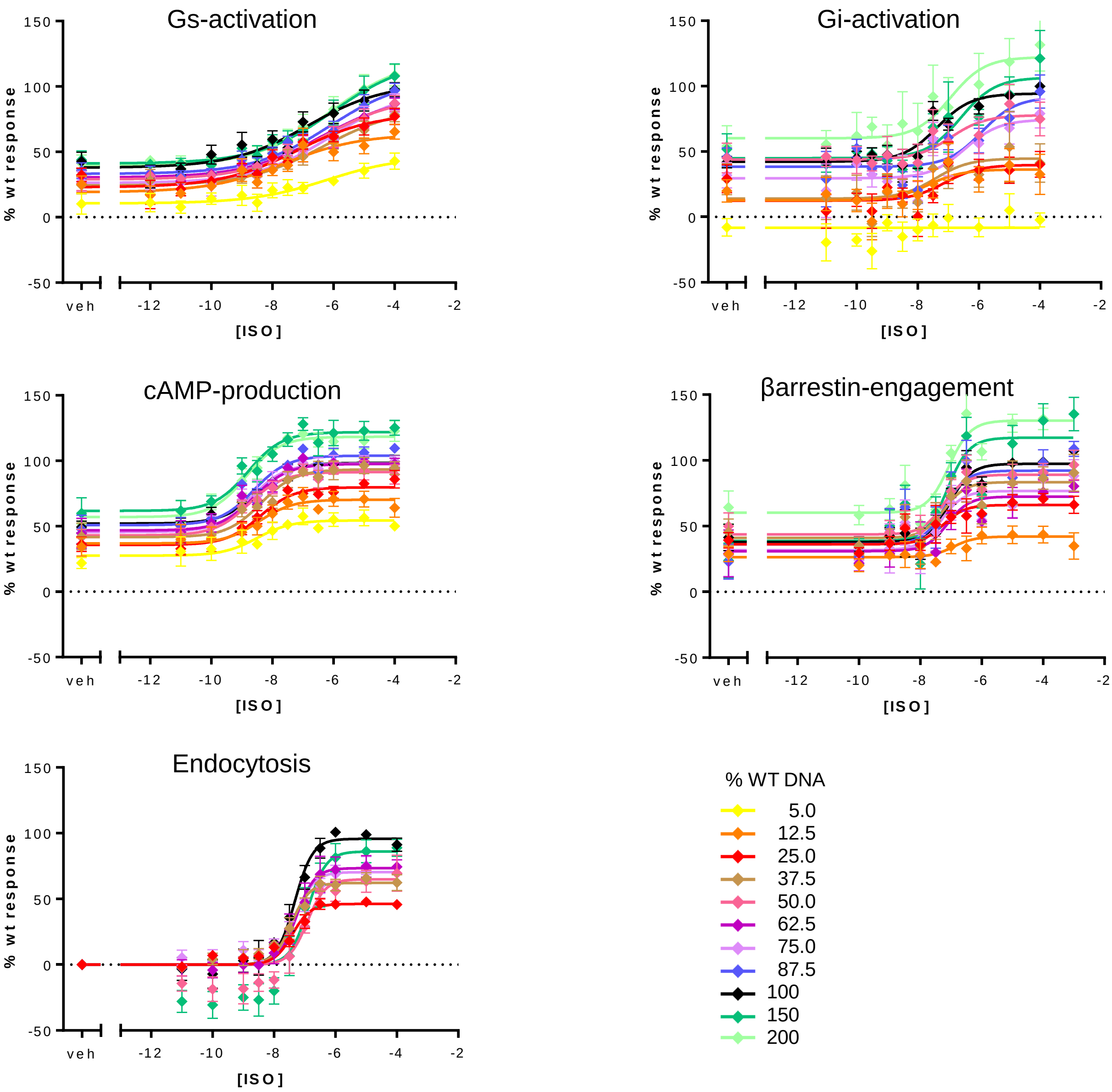

Supplementary Figure 3 | Correction for the variation in cell surface expression level. In order to correct for the difference in cell surface expression of the different β_2AR variants, concentration response curves for WT β_2AR using 5% to 150% of DNA-amount used for the variants were generated for each assay (Gs-activation, Gi activation, cAMP production, βarrestin recruitment and endocytosis) and cell surface expression measured (**Fig. 3a**). The signaling parameters (basal, maximal and ISO-induced activity, pEC_{50} and $\log T/K_A$) were then determined for each % of WT β_2AR DNA for each assay and their correlation to expression level calculated (**Fig. 3b**).

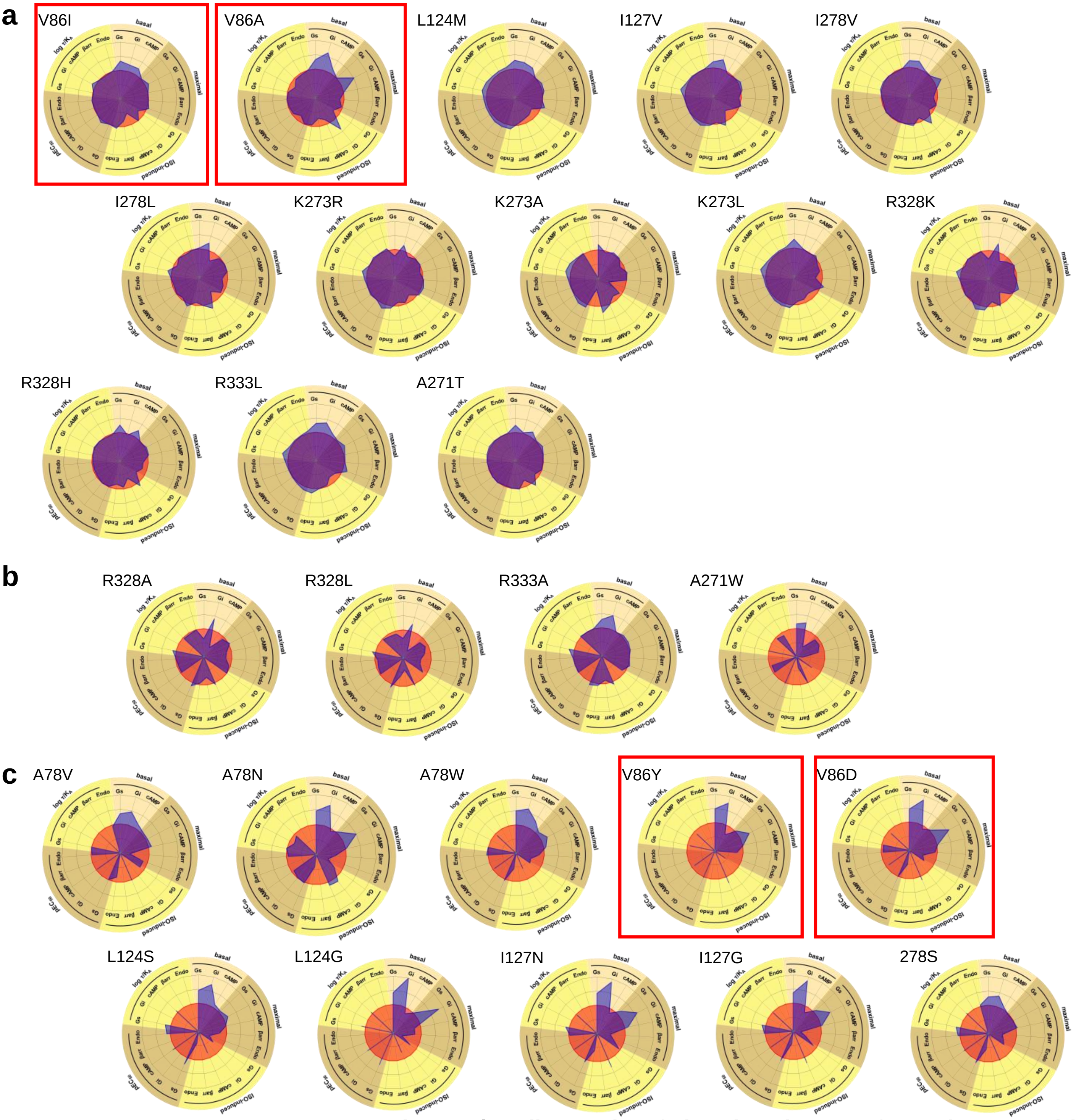

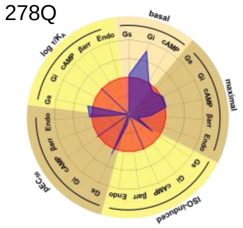

Supplementary Figure 4 | Radial graphs of signaling signature for each mutant. (a) cluster 1, **(b)** cluster 2 and **(c)** cluster 3. Signaling signature for four V86-mutations with increasing EA score are highlighted by red squares. The low EA-score mutation (V86I) slightly increases basal G-protein activation and slightly reduces ISO-stimulated Gs-activation and βarrestin2-engagement. The medium low EA-score mutation (V86A) increases basal G-protein activity, as well as maximal and ISO-induced Gi activation. ISO-stimulated Gs-activation and βarrestin2-engagement are slightly reduced. The medium high EA-score mutation (V86Y) increases basal G-protein activation and Gi maximal activity. Additionally, a decrease in ISO-stimulated Gs-activation and no response to ISO in Gi activation, βarrestin2-engagement and endocytosis are observed. The high EA-score mutation (V86D) shows a similar phenotype to V86Y, but slightly more pronounced. Data is shown as normalized difference (mut-WT/mut+WT). WT is shown in red as reference and each receptor variant is shown in blue.

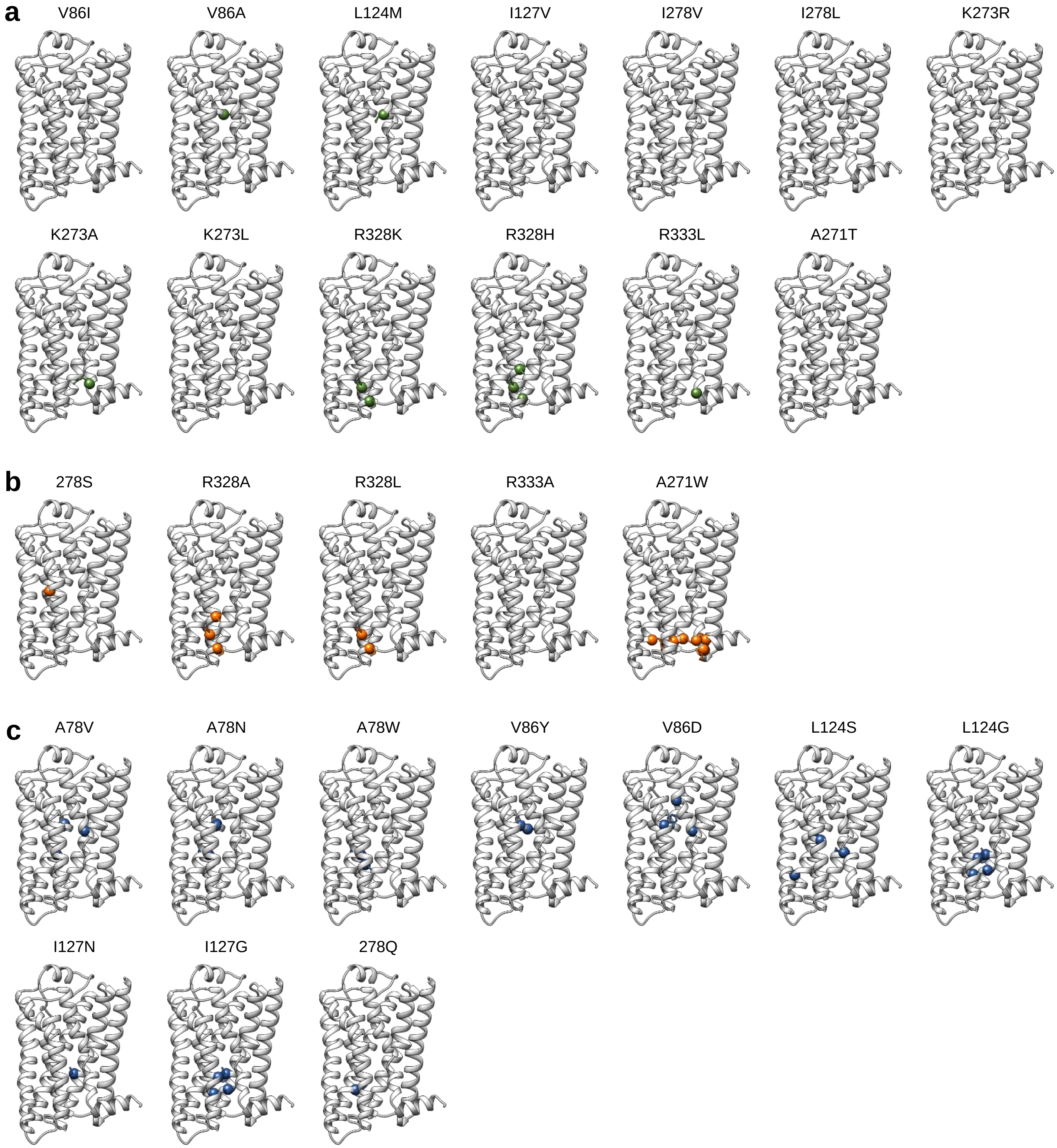

Supplementary Figure 5 | Predicted changes within 4.5 Å of each mutated position on the inactive conformation. (**a**) cluster 1, (**b**) cluster 2 and (**c**) cluster 3 are mapped on the inactive conformation (pdb: 2RH1).

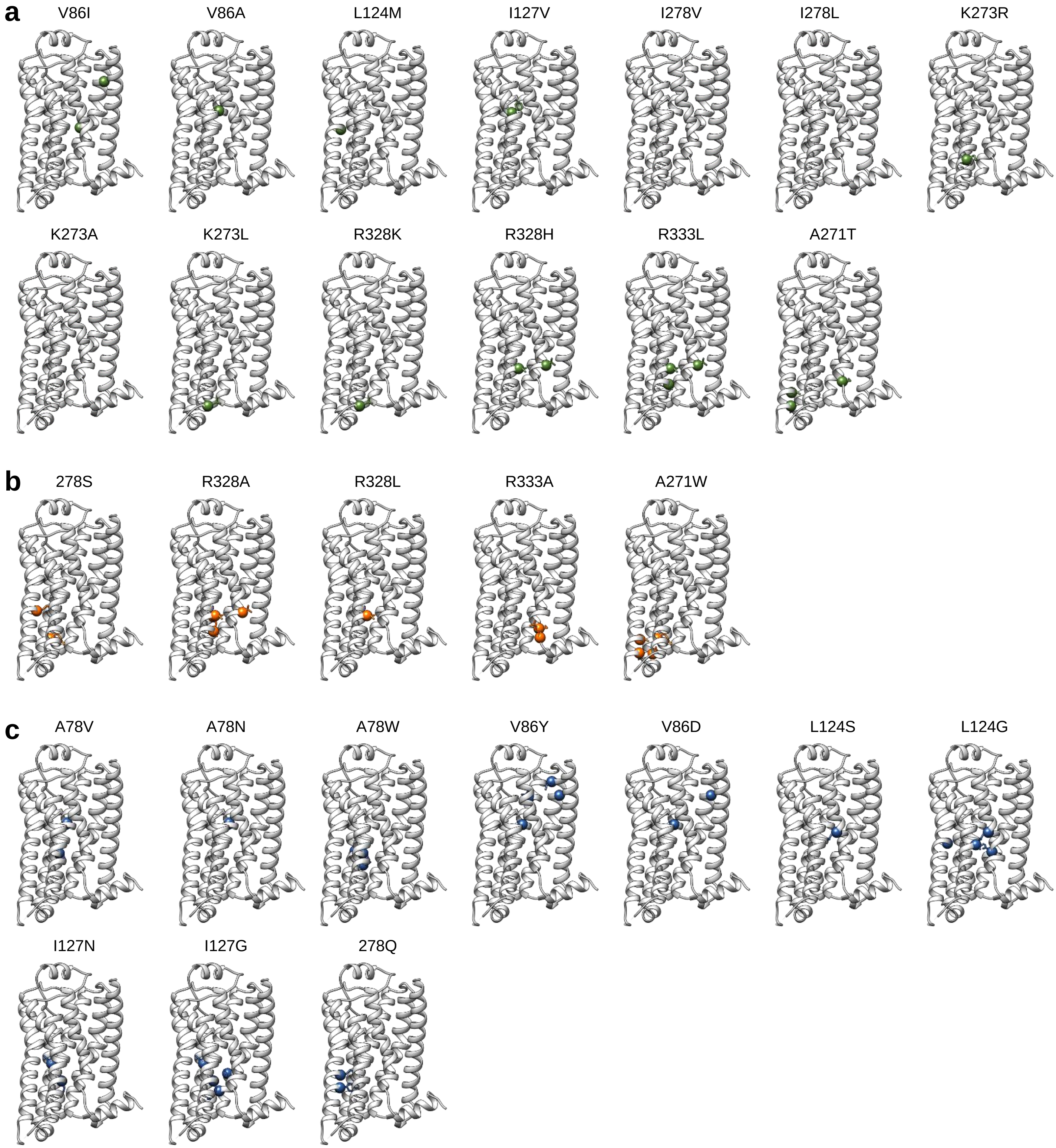

Supplementary Figure 6 | Predicted changes within 4.5 Å of each mutated position on the active conformation. (**a**) cluster 1, (**b**) cluster 2 and (**c**) cluster 3 are mapped on the active conformation (pdb: 4LDE).

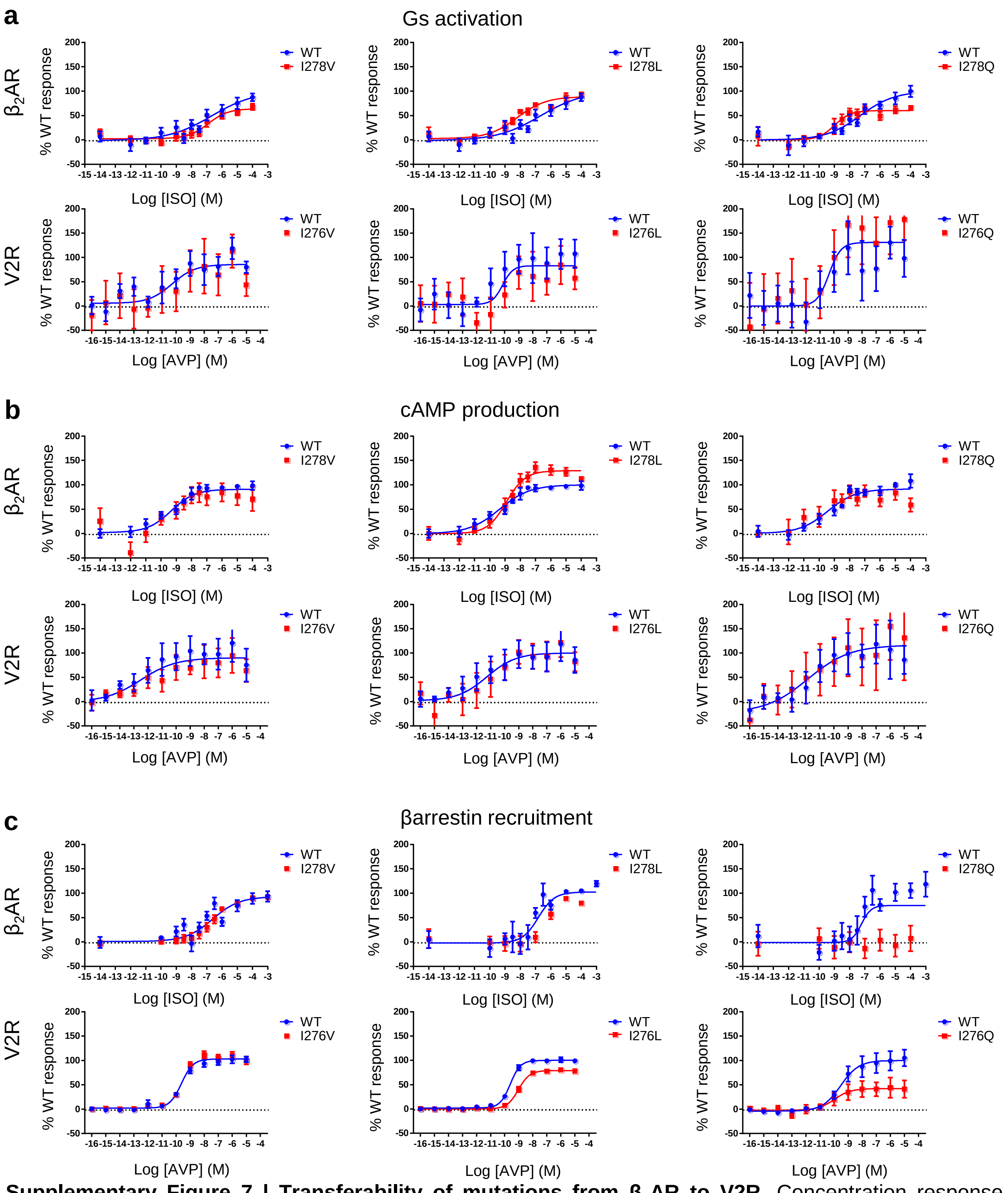

Supplementary Figure 7 | Transferability of mutations from β$_2$AR to V2R. Concentration response curves for (**a**) Gs activation, (**b**) cAMP production and (**c**) βarrestin recruitment of I$^{6.40}$ position in β$_2$AR and V2R. Mutants are shown in red and an equivalent concentration of WT receptor in blue. Data show that the mutation is transferable from the β$_2$AR to the V2R with similar effects. I276V/L doesn't or slightly affect Gαs activation, cAMP production and βarrestin recruitment whereas the I276Q variant affects βarrestin recruitment without affecting the two other pathways. Data shown are the average ±s.e.m. of at least 3 independent experiments performed in duplicate.

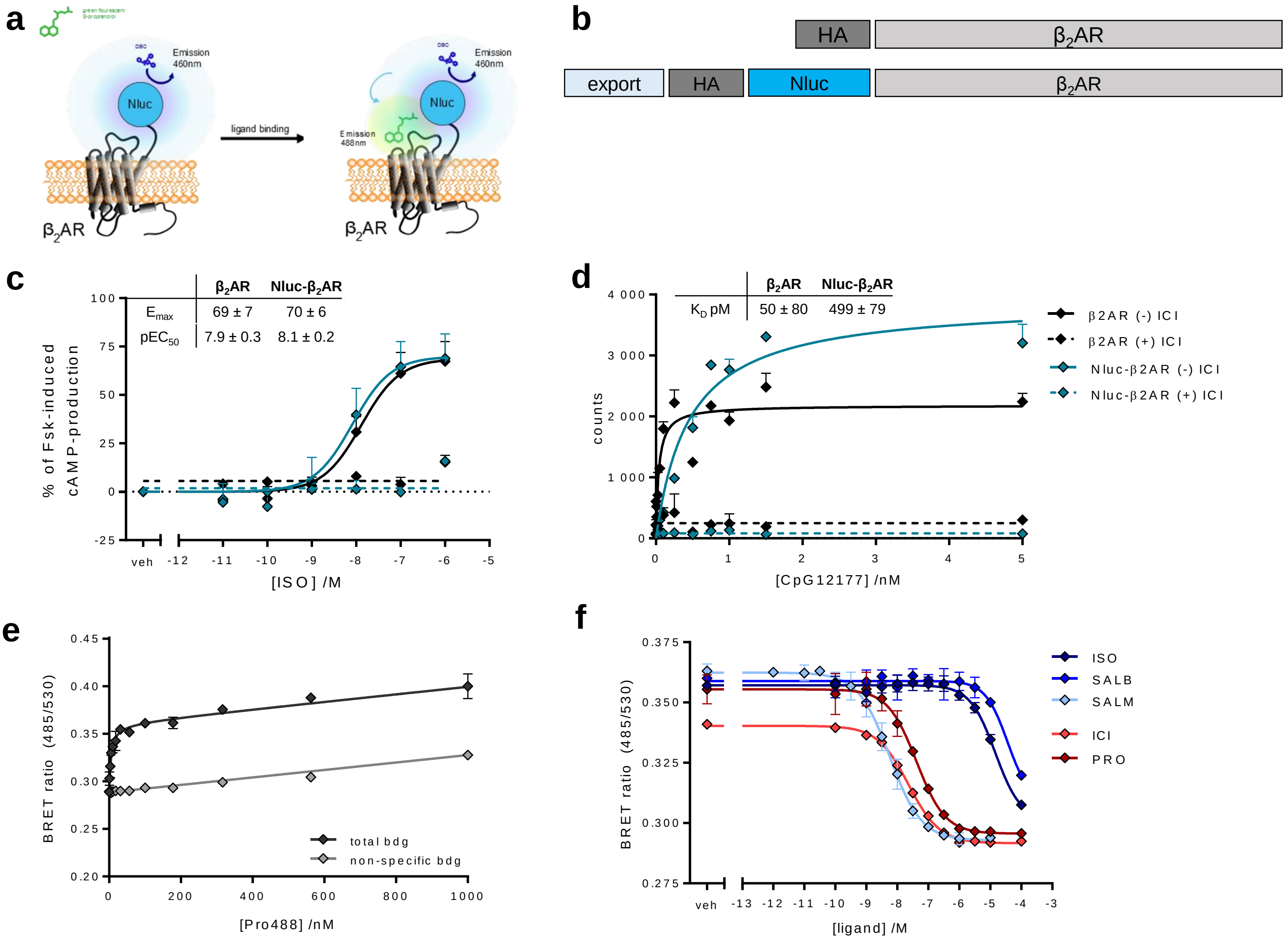

Supplementary Figure 8 | BRET-based binding assay. (**a**) Principle of BRET-based binding assay. (**b**) Schematic representation of receptor constructs. The receptor was modified at the N-terminus as indicated. (**c**) ISO-induced cAMP-production is not changed in Nluc-β_2AR. (**d**) The Kd of the radioligand CpG12177 to Nluc-β_2AR is slightly increased. (**e**) Total and non-specific binding of (S)-Propranolol-green was determined by saturation experiments. (**f**) For several β_2AR ligands the pK_i can be determined in competition with 100nM (S)-Propranolol-green and fitting with “One-site - Fit Ki” for competition binding in Prism. All data shown are representatives of at least 2 independent experiments performed in duplicate.

Supplementary Table 1 | Evolutionary importance (ET), distance to the ligand and to the nearest water molecule and substituted amino acid with evolutionary action score (EA) for each mutational target.

	ET score	dist to ligand Å	dist to water Å	Mutation (EA-score)
**A78**$^{2.49}$	24	10.4	6.4	V (58)<br>N (70)<br>W (58)
**V86**$^{2.57}$	33	5.9	9.3	I (26)<br>A (53)<br>Y (73)<br>D (90)
**L124**$^{3.43}$	7	12.7	5.5	M (55)<br>S (97)<br>G (99)
**I127**$^{3.46}$	7	17.7	7.4	V (59)<br>N (94)<br>G (99)
**I278**$^{6.40}$	18	15.7	2.9	V (41)<br>L (49)<br>S (89)<br>Q (90)
**K273**$^{6.35}$	37	23.9	9.8	R (27)<br>L (69)<br>A (66)
**R328**$^{7.55}$	39	25.1	9.3	K (27)<br>H (42)<br>L (63)<br>A (63)
**R333**$^{8.51}$	n. d.	30.7	10.2	L (n. d.)<br>A (n. d.)
**A271**$^{6.33}$	31	25.7	6.0	T (54)<br>W (78)

n. d., not determined; Ballesteros-Weinstein numbering[42] is given in superscript

Supplementary Table 2 | Signaling parameters of Gs-activation of 28 β_2AR variants.

	basal	max	ISO	pEC_{50}	logT/Ka
**WT** (13)	46 ± 2	104 ± 4	58 ± 4	7.2 ± 03	6.7 ± 0.2
**A78V** (6)	57 ± 2	100 ± 5	43 ± 4	6.4 ± 0.4	5.9 ± 0.4
**A78N** (5)	62 ± 2	93 ± 4	31 ± 4	6.9 ± 0.5	6.1 ± 0.5
**A78W** (5)	67 ± 2	89 ± 4	22 ± 4	7.2 ± 0.8	6.0 ± 0.7
**V86I** (6)	62± 3	102 ± 7	40 ± 7	6.3 ± 0.7	5.6 ± 0.3
**V86A** (6)	56 ± 2	93 ± 5	37 ± 5	6.3 ± 0.5	5.5 ± 0.4
**V86Y** (5)	63 ± 3	78 ± 7	15 ± 7	6.7 ± 2.0	4.9 ± 1.1
**V86D** (5)	57 ± 2	81 ± 5	24 ± 5	6.7 ± 0.9	5.3 ± 0.7
**L124M** (3)	70 ± 4	112 ± 4	42 ± 5	8.4 ± 0.6	8.4 ± 0.6
**L124S** (3)	61 ± 8	86 ± 3	25 ± 8	10.7 ± 1.4	8.6 ± 1.0
**L124G** (3)	38 ± 6	63 ± 3	25 ± 6	10.1 ± 1.2	7.6 ± 0.9
**I127V** (3)	61 ± 3	113 ± 3	51 ± 4	8.4 ± 0.3	8.4 ± 0.4
**I127N** (3)	58 ± 3	107 ± 4	49 ± 5	7.9 ± 0.4	7.5 ± 0.4
**I127G** (3)	55 ± 3	98 ± 4	44 ± 4	8.2 ± 0.5	7.6 ± 0.4
**I278V** (6)	61 ± 3	115 ± 7	55 ± 7	6.3 ± 0.5	6.4 ± 0.3
**I278L** (3)	40 ± 3	105 ± 4	65 ± 4	8.1 ± 0.3	7.6 ± 0.3
**I278S** (3)	72 ± 3	111 ± 3	39 ± 4	8.2 ± 0.5	8.0 ± 0.5
**I278Q** (3)	32 ± 4	71 ± 3	40 ± 4	9.1 ± 0.6	7.6 ± 0.5
**K273R** (3)	18 ± 4	86 ± 3	68 ± 4	9.1 ± 0.3	8 2 ± 0.3
**K273L** (3)	53 ± 4	116 ± 5	62 ± 6	8.2 ± 0.4	8.4 ± 0.3
**K273A** (3)	70 ± 4	124 ± 5	54 ± 5	8.0 ± 0.5	8.9 ± 0.5
**R328K** (3)	19 ± 3	85 ± 2	65 ± 3	9.2 ± 0.3	8.2 ± 0.3
**R328H** (6)	64 ± 2	104 ± 7	40 ± 6	6.3 ± 0.7	5.6 ± 0.3
**R328L** (3)	15 ± 3	82 ± 3	67 ± 3	8.2 ± 0.2	7.4 ± 0.3
**R328A** (3)	22 ± 3	80 ± 3	58 ± 4	8.3 ± 0.3	7.3 ± 0.3
**R333L** (3)	63 ± 3	119 ± 4	56 ± 5	8.2 ± 0.4	8.6 ± 0.4
**R333A** (3)	70 ± 3	120 ± 4	49 ± 4	8.4 ± 0.4	9.0 ± 0.5
**A271T** (6)	62 ± 3	116 ± 9	54 ± 9	6.0 ± 0.6	6.2 ± 0.3
**A271W** (3)	60 ± 3	101 ± 5	40 ± 5	6.9 ± 0.6	5.9 ± 0.4

Basal, maximal (max) and ISO-induced (ISO) activity are given as % of WT maximal activity. Potency (pEC50) is expressed as -log EC50 M. Signaling efficiency (logT/Ka) was calculated as described in Methods. All data are given as average ±s.e.m. of at least 3 independent experiments performed in duplicate. n for each receptor variant is given in brackets. Note: data in this table are not corrected for expression levels.

Supplementary Table 3 | Signaling parameters of Gi-activation of 28 β_2AR variants.

	basal	max	ISO	pEC_{50}	logT/Ka
**WT** (22)	28 ± 3	96 ± 5	68 ± 5	7.3 ± 0.2	6.7 ± 0.2
**A78V** (4)	67 ± 3	67 ± 3	0	n. d.	n. d.
**A78N** (3)	101 ± 6	142 ± 14	41 ± 14	6.6 ± 0.9	4.1 ± 0.6
**A78W** (4)	85 ± 4	85 ± 4	0	n. d.	n. d.
**V86I** (7)	66 ± 13	115 ±13	49 ± 17	8.2 ± 0.9	7.5 ± 0.4
**V86A** (3)	76 ± 5	148 ±53	72 ± 52	4.5 ± 1.1	3.9 ± 0.4
**V86Y** (3)	79 ± 4	79 ± 4	0	n. d.	n. d.
**V86D** (3)	147 ± 7	147 ± 7	0	9.9 ± 2.5	n. d.
**L124M** (4)	76 ± 8	110 ± 4	34 ± 8	8.5 ± 0.6	7.7 ± 1.1
**L124S** (3)	39 ± 4	39 ± 4	0	n. d.	n. d.
**L124G** (3)	54 ± 3	54 ± 3	0	n. d.	n. d.
**I127V** (3)	81 ± 6	105 ± 5	24 ± 7	7.7 ± 0.7	6.6 ± 1.5
**I127N** (3)	120 ± 5	120 ± 5	0	n. d.	n. d.
**I127G** (4)	74 ± 3	74 ± 3	0	n. d.	n. d.
**I278V** (5)	95 ± 9	196 ± 17	100 ± 18	6.6 ± 0.5	6.6±0.3
**I278L** (4)	52 ± 3	72 ± 6	19 ± 7	5.9 ± 0.9	4.7 ± 1.7
**I278S** (4)	75 ± 3	75 ± 3	0	n. d.	n. d.
**I278Q** (3)	25 ± 3	25 ± 3	0	n. d.	n. d.
**K273R** (4)	60 ± 6	92 ± 7	31 ± 8	6.9 ± 0.6	5.8 ± 0.9
**K273L** (4)	28 ± 9	57 ± 5	29 ± 10	8.7 ± 0.9	7.6 ± 1.3
**K273A** (4)	54 ± 8	88 ± 6	34 ± 9	8.0 ± 0.6	7.0 ± 0.9
**R328K** (4)	82 ± 5	105 ± 8	24± 9	6.3± 0.9	5.3 ± 1.3
**R328H** (5)	47 ± 6	121 ± 10	74 ± 11	6.8 ± 0.4	6.3 ± 0.4
**R328L** (4)	107 ± 3	107 ± 3	0	n. d.	n. d.
**R328A** (5)	94 ± 4	94 ± 4	0	n. d.	n. d.
**R333L** (4)	64 ± 5	64 ± 5	0	n. d.	n. d.
**R333A** (4)	81 ± 7	108 ± 6	28 ± 9	7.6 ± 0.8	6.6 ± 1.1
**A271T** (5)	61 ± 9	141 ±15	80 ± 16	6.9 ± 0.5	6.6 ± 0.4
**A271W** (4)	108 ± 5	108 ± 5	0	n. d.	n. d.

Basal, maximal (max) and ISO-induced (ISO) activity are given as % of WT maximal activity. Potency (pEC50) is expressed as -log EC50 M. Signaling efficiency (logT/Ka) was calculated as described in Methods. All data is given as average ±s.e.m. of at least 3 independent experiments performed in duplicate. n for each receptor variant is given in brackets. Note: data in this table is not corrected for expression levels. n. d., not determined

Supplementary Table 4 | Signaling parameters of cAMP-production of 28 β_2AR variants.

	basal	max	ISO	pEC_{50}	logT/Ka
**WT** (23)	58 ± 2	99 ± 1	41 ± 2	8.8 ± 0.1	8.5 ± 0.1
**A78V** (5)	60 ± 4	105±4	45 ± 6	7.8 ± 0.3	7.5 ± 0.2
**A78N** (4)	34 ± 3	85 ± 4	51 ± 4	7.3 ± 0.3	6.9 ± 0.3
**A78W** (5)	76 ± 3	93 ± 4	17 ± 5	7.5 ± 0.8	7.0 ± 0.7
**V86I** (5)	80 ± 4	100 ± 3	20 ± 4	8.3 ± 0.6	7.9 ± 0.6
**V86A** (5)	59 ± 4	90 ± 4	31 ± 5	7.7 ± 0.4	7.3 ± 0.4
**V86Y** (5)	18 ± 3	76 ± 4	57 ± 5	6.7 ± 0.2	6.3 ± 0.2
**V86D** (5)	29 ± 3	78 ± 5	49 ± 6	6.7 ± 0.3	6.3 ± 0.2
**L124M** (6)	68 ± 5	102 ± 2	34 ± 5	9.8 ± 0.4	9.6 ± 0.4
**L124S** (4)	40 ± 7	61 ± 3	21 ± 7	10.4 ± 0.9	9.5 ± 0.9
**L124G** (4)	19 ± 5	36 ± 2	17 ± 5	10.4 ± 1.2	9.3 ± 1.2
**I127V** (6)	45 ± 4	110 ± 3	65 ± 5	8.8 ± 0.2	8.7 ± 0.2
**I127N** (4)	23 ± 5	79 ± 5	57 ± 6	7.9 ± 0.3	7.5 ± 0.2
**I127G** (7)	22 ± 3	76 ± 2	54 ± 3	8.0 ± 0.2	7.6 ± 0.2
**I278V** (7)	63 ± 7	105 ± 4	43 ± 7	8.7 ± 0.3	8.4 ± 0.2
**I278L** (6)	23 ± 3	90 ± 3	68 ± 4	8.3 ± 0.2	8.1 ± 0.2
**I278S** (6)	56 ± 5	107 ± 3	51 ± 5	9.2 ± 0.3	9.0 ± 0.2
**I278Q** (4)	35 ± 5	70 ± 3	35 ± 6	9.1 ± 0.5	8.5 ± 0.5
**K273R** (6)	39 ± 5	87 ± 2	48 ± 5	9.3 ± 0.3	8.9 ± 0.3
**K273L** (9)	48 ± 4	96 ± 2	47 ± 5	9.2 ± 0.3	8.9 ± 0.2
**K273A** (7)	53 ± 6	98 ± 3	45 ± 7	9.2 ± 0.3	9.0 ± 0.2
**R328K** (6)	32 ± 4	94 ± 3	63 ± 5	8.6 ± 0.2	8.4 ± 0.2
**R328H** (5)	91 ± 5	120 ± 4	29 ± 6	7.9 ± 0.5	8.3 ± 0.6
**R328L** (3)	12 ± 3	70 ± 3	58 ± 3	7.9 ± 0.3	7.5 ± 0.2
**R328A** (4)	10 ± 2	73 ± 2	64 ± 3	8.0 ± 0.3	7.6 ± 0.3
**R333L** (9)	43 ± 4	97 ± 3	54 ± 4	8.9 ± 0.2	8.7 ± 0.2
**R333A** (6)	60 ± 5	105 ± 3	45 ± 5	9.0 ± 0.2	8.8±0.2
**A271T** (7)	81 ± 5	117 ± 4	36 ± 6	8.5 ± 0.3	8.7±0.3
**A271W** (8)	15 ± 3	76 ± 3	61 ± 4	7.6 ± 0.2	7.2±0.1

Basal, maximal (max) and ISO-induced (ISO) activity are given as % of WT maximal activity. Potency (pEC50) is expressed as -log EC50 M. Signaling efficiency (logT/Ka) was calculated as described in Methods. All data is given as average ±s.e.m. of at least 3 independent experiments performed in duplicate. n for each receptor variant is given in brackets. Note: data in this table is not corrected for expression levels.

Supplementary Table 5 | Signaling parameters of βarrestin2-engagement of 28 β_2AR variants.

	max	ISO	pEC_{50}	logT/Ka
**WT** (27)	98 ± 2	69 ± 3	7.0 ± 0.1	7.0 ± 0.1
**A78V** (6)	15 ± 3	0	n. d.	n. d.
**A78N** (6)	31 ± 4	0	n. d.	n. d.
**A78W** (4)	49 ± 2	0	n. d.	n. d.
**V86I** (5)	110 ± 5	44 ± 6	6.9 ± 0.3	6.5 ± 0.2
**V86A** (5)	52 ± 3	20 ± 4	6.6 ± 0.4	6.2 ± 0.6
**V86Y** (4)	38 ± 3	0	n. d.	n. d.
**V86D** (9)	51 ± 2	0	n. d.	n. d.
**L124M** (4)	84 ± 4	43 ± 7	8.2 ± 0.3	8.1 ± 0.5
**L124S** (4)	28 ± 3	0	n. d.	n. d.
**L124G** (4)	39 ± 2	0	n. d.	n. d.
**I127V** (4)	73 ± 5	42 ± 6	7.1 ± 0.3	6.9 ± 0.4
**I127N** (4)	15 ± 2	0	n. d.	n. d.
**I127G** (4)	7 ± 1	0	n. d.	n. d.
**I278V** (6)	105 ± 5	96 ± 6	6.6 ± 0.1	6.6 ± 0.1
**I278L** (4)	59 ± 5	49 ± 6	6.3 ± 0.2	6.1 ± 0.4
**I278S** (5)	34 ± 3	0	n. d.	n. d.
**I278Q** (4)	44 ± 3	0	n. d.	n. d.
**K273R** (4)	87 ± 5	35 ± 6	7.3 ± 0.4	7.1 ± 0.6
**K273L** (4)	82 ± 4	70 ± 6	7.4 ± 0.2	7.3 ± 0.2
**K273A** (8)	53 ± 4	38 ± 6	7.8 ± 0.3	7.6 ± 0.4
**R328K** (4)	95 ±5	38 ± 6	6.5 ± 0.4	6.4 ± 0.5
**R328H** (9)	87 ± 6	65 ± 7	6.5 ± 0.2	6.4 ± 0.1
**R328L** (4)	71 ± 4	37 ± 4	6.4 ± 0.3	6.2 ± 0.6
**R328A** (4)	60 ± 4	20 ± 4	6.1 ± 0.4	5.7 ± 0.9
**R333L** (8)	77 ± 5	32 ± 7	7.1 ± 0.4	6.9 ± 0.6
**R333A** (4)	89 ± 3	45 ± 5	7.4 ± 0.2	7.3 ± 0.3
**A271T** (6)	128 ± 6	85 ± 7	6.9 ± 0.2	6.9 ± 0.1
**A271W** (4)	14 ± 3	15 ± 3	6.1 ± 0.4	5.4 ± 1.2

Basal, maximal (max) and ISO-induced (ISO) activity are given as % of WT maximal activity. Potency (pEC50) is expressed as -log EC50 M. Signaling efficiency (logT/Ka) was calculated as described in Methods. All data is given as average ±s.e.m. of at least 3 independent experiments performed in duplicate. n for each receptor variant is given in brackets. Note: data in this table is not corrected for expression levels. n. d., not determined

Supplementary Table 6 | Signaling parameters of endocytosis of 28 β_2AR variants.

	max	ISO	pEC$_{50}$	logT/Ka
**WT** (19)	96 ± 3	96 ± 3	7.5 ± 0.1	7.5 ± 0.1
**A78V** (7)	41 ± 4	41 ± 4	6.6 ± 0.2	6.5 ± 0.2
**A78N** (4)	13 ± 2	13 ± 2	6.3 ± 0.3	n. d.
**A78W** (5)	18 ± 3	18 ± 3	7.8 ± 0.3	n. d.
**V86I** (4)	92 ± 3	92 ± 3	7.3 ± 0.1	7.3 ± 0.1
**V86A** (7)	54 ± 3	54 ± 3	7.1 ± 0.1	7.0 ± 0.2
**V86Y** (8)	-5 ± 1	0	n. d.	n. d.
**V86D** (5)	15 ± 3	15 ± 3	6.3 ± 0.3	n. d.
**L124M** (7)	117 ± 5	117 ± 5	8.6 ± 0.1	8.6 ± 0.1
**L124S** (6)	12 ± 2	12 ± 2	9.2 ± 0.5	n. d.
**L124G** (6)	6 ± 1	0	n. d.	n. d.
**I127V** (6)	84 ± 5	84 ± 5	7.6 ± 0.1	7.6 ± 0.1
**I127N** (6)	17 ±2	17 ± 2	6.6 ± 0.3	n. d.
**I127G** (6)	12 ±2	12 ± 2	7.5 ± 0.5	n. d.
**I278V** (8)	137 ± 8	137 ± 8	6.8 ± 0.1	6.8 ± 0.1
**I278L** (6)	59 ± 5	59 ± 5	7.0 ± 0.2	6.9 ± 0.2
**I278S** (8)	28 ± 2	28 ± 2	8.2 ± 0.2	7.9 ± 0.4
**I278Q** (6)	10 ± 2	10 ± 2	8.0 ± 0.5	n. d.
**K273R** (5)	105 ± 6	105 ± 6	8.1 ± 0.1	8.2 ± 0.1
**K273L** (4)	19 ± 4	19 ± 4	8.0 ± 0.5	n. d.
**K273A** (3)	134 ± 7	134 ± 7	8.2 ± 0.1	8.2 ± 0.1
**R328K** (5)	120 ± 8	120 ± 8	7.3 ± 0.2	7.2 ± 0.1
**R328H** (5)	92 ± 6	92 ± 6	6.7 ± 0.1	6.7 ± 0.1
**R328L** (5)	107 ± 6	107 ± 6	7.0 ± 0.1	7.0 ± 0.1
**R328A** (8)	47 ± 5	47 ± 5	5.8 ± 0.2	5.7 ± 0.3
**R333L** (6)	67 ± 4	67 ± 4	7.5± 0.1	7.5 ± 0.2
**R333A** (6)	109 ± 7	109 ± 7	8.3± 0.2	8.4 ± 0.1
**A271T** (4)	126 ± 5	126 ± 5	7.3± 0.1	7.3 ± 0.1
**A271W** (5)	-2 ± 4	0	n. d.	n. d.

Basal, maximal (max) and ISO-induced (ISO) activity are given as % of WT maximal activity. Potency (pEC50) is expressed as -log EC50 M. Signaling efficiency (logT/Ka) was calculated as described in Methods. All data is given as average ±s.e.m. of at least 3 independent experiments performed in duplicate. n for each receptor variant is given in brackets. Note: data in this table is not corrected for expression levels. n. d., not determined

Supplementary Table 7 | Mutation similarity matrix derived from the non-negative matrix factorization/K-means clustering method

	V86I	V86A	L124M	I127V	A271T	K273R	K273A	K273L	I278V	I278L	R328K	R328H	R333L	A271W	R328A	R328L	R333A	A78V	A78N	A78W	V86Y	V86D	L124S	L124G	I127N	I127G	I278Q	I278S
V86I	1	0.998	1	0.994	1	0.965	0.997	0.999	1	0.876	0.808	1	1	0	0	0	0.142	0	0	0	0	0	0	0	0	0	0	0
V86A	0.998	1	0.998	0.992	0.998	0.963	0.995	0.997	0.998	0.874	0.806	0.998	0.998	0.002	0.002	0.002	0.144	0	0	0	0	0	0	0	0	0	0	0
L124M	1	0.998	1	0.994	1	0.965	0.997	0.999	1	0.876	0.808	1	1	0	0	0	0.142	0	0	0	0	0	0	0	0	0	0	0
I127V	0.994	0.992	0.994	1	0.994	0.971	0.991	0.995	0.994	0.882	0.814	0.994	0.994	0.006	0.006	0.006	0.148	0	0	0	0	0	0	0	0	0	0	0.003
A271T	1	0.998	1	0.994	1	0.965	0.997	0.999	1	0.876	0.808	1	1	0	0	0	0.142	0	0	0	0	0	0	0	0	0	0	0
K273R	0.965	0.963	0.965	0.971	0.965	1	0.962	0.966	0.965	0.887	0.843	0.965	0.965	0.035	0.035	0.035	0.173	0	0	0	0	0	0	0	0	0	0	0.008
K273A	0.997	0.995	0.997	0.991	0.997	0.962	1	0.996	0.997	0.879	0.811	0.997	0.997	0.003	0.003	0.003	0.145	0	0	0	0	0	0	0	0	0	0	0
K273L	0.999	0.997	0.999	0.995	0.999	0.966	0.996	1	0.999	0.877	0.809	0.999	0.999	0.001	0.001	0.001	0.143	0	0	0	0	0	0	0	0	0	0	0
I278V	1	0.998	1	0.994	1	0.965	0.997	0.999	1	0.876	0.808	1	1	0	0	0	0.142	0	0	0	0	0	0	0	0	0	0	0
I278L	0.876	0.874	0.876	0.882	0.876	0.887	0.879	0.877	0.876	1	0.83	0.876	0.876	0.124	0.124	0.124	0.26	0	0	0	0	0	0	0	0	0	0	0.018
R328K	0.808	0.806	0.808	0.814	0.808	0.843	0.811	0.809	0.808	0.83	1	0.808	0.808	0.192	0.192	0.192	0.29	0	0	0	0	0	0	0	0	0	0	0.023
R328H	1	0.998	1	0.994	1	0.965	0.997	0.999	1	0.876	0.808	1	1	0	0	0	0.142	0	0	0	0	0	0	0	0	0	0	0
R333L	1	0.998	1	0.994	1	0.965	0.997	0.999	1	0.876	0.808	1	1	0	0	0	0.142	0	0	0	0	0	0	0	0	0	0	0
A271W	0	0.002	0	0.006	0	0.035	0.003	0.001	0	0.124	0.192	0	0	1	1	1	0.857	0.001	0	0	0	0	0	0	0	0	0	0.167
R328A	0	0.002	0	0.006	0	0.035	0.003	0.001	0	0.124	0.192	0	0	1	1	1	0.857	0.001	0	0	0	0	0	0	0	0	0	0.167
R328L	0	0.002	0	0.006	0	0.035	0.003	0.001	0	0.124	0.192	0	0	1	1	1	0.857	0.001	0	0	0	0	0	0	0	0	0	0.167
R333A	0.142	0.144	0.142	0.148	0.142	0.173	0.145	0.143	0.142	0.26	0.29	0.142	0.142	0.857	0.857	0.857	1	0.002	0.001	0.001	0.001	0.001	0.001	0.001	0.001	0.001	0.001	0.163
A78V	0	0	0	0	0	0	0	0	0	0	0	0	0	0.001	0.001	0.001	0.002	1	0.999	0.999	0.999	0.999	0.999	0.999	0.999	0.999	0.999	0.834
A78N	0	0	0	0	0	0	0	0	0	0	0	0	0	0	0	0	0.001	0.999	1	1	1	1	1	1	1	1	1	0.833
A78W	0	0	0	0	0	0	0	0	0	0	0	0	0	0	0	0	0.001	0.999	1	1	1	1	1	1	1	1	1	0.833
V86Y	0	0	0	0	0	0	0	0	0	0	0	0	0	0	0	0	0.001	0.999	1	1	1	1	1	1	1	1	1	0.833
V86D	0	0	0	0	0	0	0	0	0	0	0	0	0	0	0	0	0.001	0.999	1	1	1	1	1	1	1	1	1	0.833
L124S	0	0	0	0	0	0	0	0	0	0	0	0	0	0	0	0	0.001	0.999	1	1	1	1	1	1	1	1	1	0.833
L124G	0	0	0	0	0	0	0	0	0	0	0	0	0	0	0	0	0.001	0.999	1	1	1	1	1	1	1	1	1	0.833
I127N	0	0	0	0	0	0	0	0	0	0	0	0	0	0	0	0	0.001	0.999	1	1	1	1	1	1	1	1	1	0.833
I127G	0	0	0	0	0	0	0	0	0	0	0	0	0	0	0	0	0.001	0.999	1	1	1	1	1	1	1	1	1	0.833
I278Q	0	0	0	0	0	0	0	0	0	0	0	0	0	0	0	0	0.001	0.999	1	1	1	1	1	1	1	1	1	0.833
I278S	0	0	0	0.003	0	0.008	0	0	0	0.018	0.023	0	0	0.167	0.167	0.167	0.163	0.834	0.833	0.833	0.833	0.833	0.833	0.833	0.833	0.833	0.833	1

The mutation similarity matrix derived from the 1000 replicates of the nnmf-Kmeans clustering method enables quantification of clustering robustness for k =3.
Specifically, similarities with frequencies close to 0 and 1 indicate very robust assignments as these β2AR variants always demonstrate the same non-random clustering pattern in relation to one another.
Clusters are color-coded (cluster 1 – green, cluster 2 – orange, cluster 3 – blue).

Supplementary Table 8 | Ligand binding parameters determined by BRET-based binding.

	pK_i (ISO)
**WT** (5)	6.2 ± 0.3
**A78V** (4)	5.8 ± 0.1
**A78N** (5)	5.9 ± 0.5
**A78W** (5)	6.6 ± 0.5
**V86I** (4)	6.1 ± 0.2
**V86A**	n. d.
**V86Y**	n. d.
**V86D**	n. d.
**L124M** (4)	6.3 ± 0.3
**L124S** (5)	8.1 ± 0.6
**L124G** (5)	n. d.
**I127V** (4)	6.5 ± 0.2
**I127N** (5)	7.7 ± 0.4
**I127G** (5)	6.2 ± 0.7
**I278V** (4)	5.6 ± 0.2
**I278L** (4)	7.3 ± 0.1
**I278S** (4)	7.3 ± 0.1
**I278Q** (4)	8.3 ± 0.5
**K273R** (4)	6.5 ± 0.2
**K273L** (4)	6.5 ± 0.1
**K273A** (4)	6.0 ± 0.3
**R328K** (4)	5.9 ± 0.1
**R328H** (4)	5.9 ± 0.1
**R328L** (4)	5.9 ± 0.2
**R328A** (4)	6.2 ± 0.3
**R333L** (5)	6.4 ± 0.3
**R333A** (4)	6.3 ± 0.1
**A271T** (5)	5.8 ± 0.3
**A271W** (4)	5.8 ± 0.1

The K_D of the fluorescent ligand (S)-Propranolol-green was determined by saturation binding for each variant of the β_2AR and was followed by the determination of pK_i for ISO by competition binding studies. Data is given as average ±s.e.m. of at least 3 independent experiments performed in duplicate. n for each receptor variant is given in brackets. n. d., not determined
